# The effect of isocaloric, energy-restrictive, KETOgenic diet on metabolism, inflammation, nutrition deficiencies and oxidative stress in women with overweight and obesity (KETO-MINOX): Study protocol

**DOI:** 10.1371/journal.pone.0285283

**Published:** 2023-05-08

**Authors:** Natalia Drabińska, Jerzy Romaszko, Paul White

**Affiliations:** 1 Department of Chemistry and Biodynamics of Food, Institute of Animal Reproduction and Food Research of Polish Academy of Sciences, Olsztyn, Poland; 2 Department of Family Medicine and Infectious Diseases, University of Warmia and Mazury in Olsztyn, Olsztyn, Poland; 3 Department of Mathematics and Data Science, University of the West of England, Bristol, United Kingdom; PLOS: Public Library of Science, UNITED KINGDOM

## Abstract

Obesity is considered one of the biggest health problems of the 21st century, becoming a worldwide epidemic, leading to the development of many diseases and increasing the risk of premature death. The first step in reducing body weight is a calorie-restricted diet. To date, there are many different diet types available, including the ketogenic diet (KD) which is recently gaining a lot of attention. However, all the physiological consequences of KD in the human body are not fully understood. Therefore, this study aims to evaluate the effectiveness of an eight-week, isocaloric, energy-restricted, KD as a weight management solution in women with overweight and obesity compared to a standard, balanced diet with the same calorie content. The primary outcome is to evaluate the effects of a KD on body weight and composition. The secondary outcomes are to evaluate the effect of KD-related weight loss on inflammation, oxidative stress, nutritional status, profiles of metabolites in breath, which informs about the metabolic changes in the body, obesity and diabetes-associated parameters, including a lipid profile, status of adipokines and hormones. Notably, in this trial, the long-term effects and efficiency of the KD will be studied. In summary, the proposed study will fill the gap in knowledge about the effects of KD on inflammation, obesity-associated parameters, nutritional deficiencies, oxidative stress and metabolism in a single study.

**ClinicalTrail.gov registration number:** NCT05652972.

## Introduction

Over the last decades, the incidence of obesity is increasing, reaching an epidemic scale and contributing to a burden on the health system. Obesity is a disease associated with excessive fat accumulation leading to an increased risk of development of serious health risks. Obesity can be classified based on the body mass index (BMI) calculated as body weight (kg) divided by height squared (m^2^). A BMI of 25 is classified as a lower threshold of overweight, not obesity yet. Any BMI above 30 is classified as obesity and can be further divided into obesity class I (BMI 30–35 kg/m^2^), class II (BMI 30–40 kg/m^2^) and class II (BMI > 40 kg/m^2^) [1]. In 2016, World Health Organization (WHO) reported that approx. 2 billion adults can be classified as overweight, of which 650 million meet the criteria of obesity. Narrowing down to Europe, almost 50% of the population struggles with being overweight [2]. Since 2010, the number of deaths caused by obesity exceeded the mortality of undernutrition and starvation [3].

Among the causes of obesity, too high-calorie intake is considered the most important factor [4]. Western diet characterized by the overconsumption of easily accessible highly processed and rich in sugar products together with low physical activity leads to fat deposition and a high prevalence of overweight and obesity. From ancient times, the human body developed mechanisms which were supposed to protect from starvation, therefore the deposition of additional fat is easier than its reduction [5]. Obesity in adulthood may be somehow programmed already during the prenatal period by environmental, genetic and epigenetic factors [6]. Other physiological and genetic aspects affect obesity development, having an impact on calorie intake, thermogenesis, lipid utilisation and nutrient turnover [7]. The endocrine system regulates satiety and appetite *via* adipokines secreted by adipose tissue [8]. Consequently, the overaggregation of fat tissue modifies its secretory status, which then causes low-grade inflammation, leading to the development of metabolic diseases. Moreover, neurological factors, such as reward systems in the brain and prolonged stress, can be a reason for overeating leading to overweight [9]. In the last years, also the COVID-19 pandemic, isolation at home and increased stress resulted in behavioural changes resulting in weight gain and obesity [10].

Overweight increases the risk of cardiovascular diseases including heart failure and hypertension, type 2 diabetes, some types of cancer and many others [5, 11]. The severity of obesity-related diseases is closely related to body fat distribution, and in particular, to visceral localisation. Visceral fat accumulation is linked to the status of adipokines, consequently inducing chronic inflammation and metabolic disorders [12]. The main adipokines produced by visceral adipose tissue are interleukin-6 (IL-6), IL-beta, leptin, tumour necrosis factor-alpha (TNF-a), adiponectin, serum amyloid A-3 (SAA3), alpha 1-acid glycoprotein, resistin, vascular endothelial growth factor (VEGF), and many others [13, 14]. The status of adipokines is also closely related to oxidative stress (Ox). Higher concentrations of adipokines induce the production of reactive oxygen species, which then affect the production of other adipokines [15]. Excessive fat accumulation leads to an increase in serum free fatty acid (FFA) levels, which affect glucose metabolism and promotes adipose, hepatic, and muscular aggregation of fats and glucose, inducing higher mitochondrial and peroxisomal oxidation, which leads to the Ox, injury of mitochondrial DNA, and, lastly, lipotoxicity, which causes various unwanted effects of fatty acids on cellular structures [16]. Then the production of cytokines is upregulated due to the cell damage, which induces the production of reactive oxygen species and further peroxidation of lipids.

The consequences of obesity, like heart failure, metabolic syndrome and respiratory issues lead to an increased death rate and a higher risk of premature death in patients with obesity [7, 9]. Recent studies suggested that increased gut permeability in individuals with obesity may lead to an increased influx of bacterial components such as lipopolysaccharides (LPS) and other complex antigens into the circulation and, thereby, activate an immune response. Therefore, there is a strong need to try to prevent obesity and overweight development.

Caloric restriction is the most commonly used way of losing weight [17]. Generally, there is no single best nutritional strategy, the crucial factor is adherence to the diet and the maintenance of energy restriction [18, 19]. Therefore, the critical issue is which type of diet to choose to efficiently lose weight, which will be continued in the follow-up period, and most importantly, a safe diet, without causing any side effects. The success of the diet depends on consistent negative energy balance and the nutritional composition of the diet [20]. To date, the effect of diets that differ in nutritional composition such as low-calorie [21], very low-calorie [22], high-protein [23], low-fat diet [24] and intermittent fasting [25] on the health-related parameters in subjects with obesity were extensively studied. Recently, a diet used as an auxiliary therapy for non-responding epilepsy [26], which is a ketogenic diet (KD) has been proposed as a solution for obesity management. The International Food Information Council (IFIC) Foundation reported that a KD is the only diet with a trending interest, among Americans, whose motivation was losing weight (http://foodinsight.org). Despite the fact that the popularity of this diet has increased among the population and the number of individuals following KD has increased in the last few years, knowledge about the safety and effectiveness of KD for weight loss is scarce.

The KD is a high-fat and low-carbohydrate diet, which contrary to other low-carbohydrate diets, leads to a ketosis state in the body. For instance, ketosis is not observed in a low-carbohydrate diet with high protein consumption primarily because up to 58% of amino acids are glycogenic, preventing ketosis from occurring [27]. The idea of KD was to mimic the starvation state without fasting.

Previous studies with the application of KD in obesity showed a significant reduction in body weight, BMI and fat mass content after following a KD, as summarized in a recent review [28]. However, it is not confirmed that this results from the ketosis state, or simply due to the very reduced calorie intake. In most of these studies on the application of KD for obesity management, very low-calorie KD was applied, limited to just 500–800 kcal/day. It can be suggested that the observed changes resulted from the highly reduced intake, not the nutritional composition of the diet, especially since most of the studies did not include a control group to compare the effect of the different nutritional compositions of diets [29–31].

Regarding the metabolic effects, very low-calorie KD resulted in more pronounced changes in the circulating IL-6, IL-8 and metalloproteinase 2, suggesting beneficial health-related aspects [32]. Moreover, very low-calorie KD supplemented with DHA reduced the concentrations of insulin, triglycerides, total cholesterol, LDL cholesterol, C-reactive protein, TNF-α and resistin [33]. The positive effects of very low-calorie KD on lipid metabolism and diabetic-related parameters were also confirmed by other authors [34–37]. Additionally, the very low-calorie KD resulted in a decrease in blood pressure [34, 38], which can be associated with reduced body weight itself. However, all of these effects were obtained in studies with very low-calorie KD, dedicated to individuals scheduled for bariatric surgery and followed under clinical supervision. Therefore, it cannot be compared with the effect of KD with appropriate caloric intake or a slight reduction, which is followed by the individuals motivated to lose weight and inspired by social media and influencers to follow KD.

There are few studies on KD with optimal caloric intake [30, 31, 39, 40]. Most of these studies did not include a control group, making results difficult to interpret. Considering the studies on KD which included a control group, the positive effect of KD on obesity management was confirmed in a study with 45 women (25 KD, 20 Control) with obesity with ovarian or endometrial cancer [41]. After twelve weeks, the authors observed a more significant loss in visceral, android and total fat mass in the individuals following KD than in the control group, not restricted to total energy intake. The KD was also associated with lower fasting serum insulin levels in women with obesity and ovarian or endometrial cancer, as suggested by the authors, to enhance insulin sensitivity [41]. However, most of the studies with KD with optimal calorie intake focused only on body weight and composition changes and a limited number of health-related parameters [28]. Therefore there is a strong need for comprehensive studies on the physiological effects of KD in individuals with overweight, especially in terms of inflammation, nutrition and metabolic changes.

Detrimentally, it has been noted that a long-term KD has been suggested as a risk factor for kidney stones, increased circulating levels of uric acid, and osteoporosis due to low calcium intake [42]. However, it is worth underlining that the long-term KD regime is followed mainly by patients with refractory epilepsy to reduce the incidence of seizures [26]. Since diet in subjects with obesity and overweight is employed to achieve significant weight loss, short diet interventions are explainable and do not usually last more than a few weeks. Therefore, the side effects are relatively mild and include hunger, fatigue, low mood, irritability, constipation and headaches. It seems to be more important to check if the nutritional composition of the diet affects body weight after the follow-up period, even without strict diet control in between. Moreover, the very restrictive nature of KD, with a limited number of allowed food products, could result in much higher drop-outs in longer intervals, related to difficulties in maintaining the diet, mainly if the extreme reduction of calories was applied.

In summary, based on the available scientific literature it can be concluded that following a KD might be a beneficial solution for obesity management. However, the comprehensive studies that warrant the safety of using KD for bodyweight loss are limited. So far, the majority of the studies were conducted with small sample sizes and a lack of control groups. Very often, the duration of the intervention was short and the follow-up checks were not performed. Furthermore, the vast majority of studies with KD included very low-calorie KD, and it is difficult to conclude if the observed changes were due to KD or very quick body weight reduction. More randomised and controlled studies, including better monitoring of diet adherence aimed at the physiological and metabolic effects of diet composition, need to be performed. In addition, although some studies were conducted on nutritional status [40], there is a necessity to complete more studies on mineral, vitamin and nutrient intakes and their status.

### Research objective and hypothesis

Taking into account information presented in the previous section, the proposed study was designed to evaluate the effect of KD on metabolism, inflammation, nutritional status, Ox, and obesity-associated parameters in a single, randomised and controlled study. To have better control over the diet consumed by participants, the food catering optimized by the dietician will be delivered to each person daily. To the best of our knowledge, there are no completed or ongoing studies focused on the efficiency of KD in a randomised design, with a properly balanced control group, with a homogenised diet provided to all participants during the whole intervention and analysing many parameters in a single study.

Based on current knowledge [28], and our pilot study with an animal model [43], we hypothesise that the energy-restricted KD will be more efficient in weight reduction and decreasing low-grade inflammation and oxidative stress parameters in women with overweight and obesity than a standard diet with the same calorie restriction.

This study aims to evaluate the effectiveness of an eight-week, isocaloric, energy-restricted, ketogenic diet as a weight management solution in women with overweight and obesity compared to a standard, balanced diet with the same calorie content.

The primary aim is:

to evaluate the effects of a KD on body weight and composition

The secondary aims are:

1. to evaluate the effect of KD-related weight loss on the inflammation and Ox in women with overweight and obesity,
2. to evaluate the effect of KD-related weight loss on the nutritional status of the human subjects,
3. to evaluate the effects of KD-related weight loss on the profiles of metabolites in breath, which informs about the metabolic changes in the body,
4. to evaluate the effect of KD-related weight loss on the obesity and diabetes-associated parameters, including a lipid profile, status of adipokines, hormones,
5. to evaluate the long-term effects and efficiency of the KD.

### Participant selection

Recruitment of study participants will be performed using announcements on social media (Facebook, Instagram) and by local press and radio. The time planned for the recruiting is expected not to exceed six months. Previous experiences with human studies [44] showed that the motivation of the study participant to take part is crucial to successful trial completion. Therefore, the participants are offered the free, planned weight-loss program, which does not require cooking the meals for eight weeks, with meals balanced by a dietician and delivered to the house daily, at a time convenient to participants. The nutritional intervention is planned for the late-spring/early-summer months since, in general, women are more motivated to lose weight before the summer [45].

**The inclusion criteria are** women, age 18–45; overweight or obesity type I (BMI 25.5–35); motivated to lose weight and motivated and willing to participate in a nutritional intervention trial.

**The exclusion criteria are** pregnancy, childbearing age not using contraceptives, breastfeeding, type 1 or type 2 diabetes, any chronic diseases requiring pharmacotherapy; participation in other clinical trials; severe obesity (BMI > 35), and any diagnosed psychiatric disorders.

### Sample size

On trial opening, the first 80 women meeting eligibility criteria who also do not meet any of the exclusion criteria will be selected. The focus on only female subjects was based on eliminating biological sex as a factor that could affect the results. Moreover, it is scientifically proven that women have better trust in healthy nutrition and more engagement and compliance in dietary programs [46, 47], thus a lower number of drop-outs is expected. At the end of the intervention, dietary efficacy is projected to be between the equivalent of one dress size to one and a half dress sizes, equating to a weight loss of approximately 5kg. For BMI between 28 and 35, the standard deviation of weight is approximately 5kg equating to an intra-group standardised effect of 1. However, a downward revision in standardised effect size for a between groups comparions for an intent-to-treat analysis Analysis of Covariance (ANCOVA) would indicate that n = 32 women per group would have at least 80% power to detect a standardised difference of Cohen’s d = 0.5 (alpha = 0.05, two-sided) assuming a pre- post- correlation in weight of 0.7. Inflation of sample sizes to account for loss to follow-up would indicate n = 40 per group would provide a safety margin for a comparison between group means. This sample size is conservatively consistent with Mohorko et al. [39]. The sample size will support within-groups, between-groups, and whole-group analyses for the secondary research questions.

### Ethical aspects

The study participants will be informed about the potential benefits and risks of the dietary intervention, and they will sign an informed consent form during the enrolment visit. The information meeting is planned to explain all the details of the trial and also to prepare participants for the potential, initial side effects of ketosis (“keto flu”–headaches, fatigue, tiredness, etc.), which can be experienced for a few first days and the solutions to mitigate it will be provided. Moreover, an effort to counsel subjects not to increase their calorie intake via supper, snacks, etc. and to fill the research diary honestly will be clarified. The participants will be advised not to introduce any additional physical activity, and that any activity (if any) has to be noted in the study diary. The experimental design and procedures were approved by the Bioethics Committee of the Faculty of Medical Sciences of the University of Warmia and Mazury in Olsztyn (agreement No: 25/2022 from 27^th^ of October 2022). The study was registered at http://www.clinicaltrials.gov (registration number NCT05652972).

### Randomization and blinding

The study participants will be randomly divided into two experimental groups. Stratified randomisation will be performed based on age and BMI. The KD group will receive a 1700 kcal ketogenic diet (fat: protein: carbohydrate ratio of 70:20:10), while the control group will receive a 1700 kcal standard balanced diet (fat: protein: carbohydrate ratio 20:30:50). Both participants and the researchers will not be blinded due to character of the intervention.

### Dietary intervention

Diets will be prepared by the outsourcing company that specialized in daily catering for losing weight. The dietician will prepare both diets to balance them in terms of the nutritional value, macro- and microelements. A KD will be based mainly on fats derived from plants, fish, and nuts (considered as “healthy KD”). The diets will be followed for 8 weeks and to be sure about the diet composition, and adherence, the meal catering consisting of 5 meals (breakfast, snack, lunch, snack II and dinner) will be delivered to each participant in the morning, every day for the whole study period. Therefore, the homogeneity of the diet will be maintained, removing the diet variation as a factor affecting the results. The 8-week intervention was selected to see the changes in the metabolic state, body weight, and inflammatory markers and avoid the unnecessary extension of the intervention associated with a higher dropout rate. Moreover, an 8-week diet plan is a common duration followed by women affected by social media.

### Sample collection

There will be 3 sample collection points for each participant during the nutritional intervention: at baseline (T0), after four weeks (T1) and after eight weeks (T2). Because of the relatively high number of participants, to avoid long queues and irritation of participants, the check-up visits will be divided into four days a week. Check-up visits will be carried out in the Pantamed Primary Care Unit under the medical doctor’s supervision and qualified nurses. Moreover, after one year (T3), the check-up visit is planned to evaluate the regain of body weight, the nutritional habits, and the potential long-term effects of this intervention, focusing on obesity, diabetes, and low-grade inflammation-associated parameters.

During each check-up visit (T0-T3), in the overnight-fasting participants, height, body weight, body composition, as well as the waist and hips circumference will be measured, the check-up made by the medical doctor, including systolic, diastolic and pulse pressure, will be performed and the samples will be collected. BMI will be calculated based on body mass [kg] divided by the square of body height [m] and expressed in kg/m^2^. The body weight and composition will be measured using TANITA Body Composition Analyser, while the waist circumference will be measured using a standard measurement tape.

The participants who will not follow the diet for more than 80% of the trial period or will initiate the pharmacological therapy prescribed by the physician during the intervention will be eliminated from the study.

### Analysis of haematological and biochemical blood parameters

Qualified nurses will collect blood samples after the overnight fasting. Four vials of blood will be collected from each participant to be sure that the appropriate amount of samples will be collected: 1) 1.6 mL into a vacuum tube containing EDTA for hematologic analysis; 2) 5 mL into a vacuum tube for biochemical serum analyses run by Diagnostic Laboratory; 3 and 4) 2 vials of 5 mL into a vacuum tube for serum analyses run in the Institute of Animal Reproduction and Food Research Polish Academy of Sciences. Exhaled breath condensate samples will be collected using commercial sampling tubes (RTubes), which allow for the collection of approx. 3 mL condensate within 10 minutes. All the samples will be stored in ice and transported to the laboratories of the Institute of Animal Reproduction and Food Research of the Polish Academy of Sciences as soon as possible, where the vials with blood will be centrifuged at 3500 rpm for 10 min. The serum and condensate samples will be aliquoted and stored until analyses either in the fridge (4°C) or an ultra-freezer (-80°C), as appropriate.

**Fig 1:**
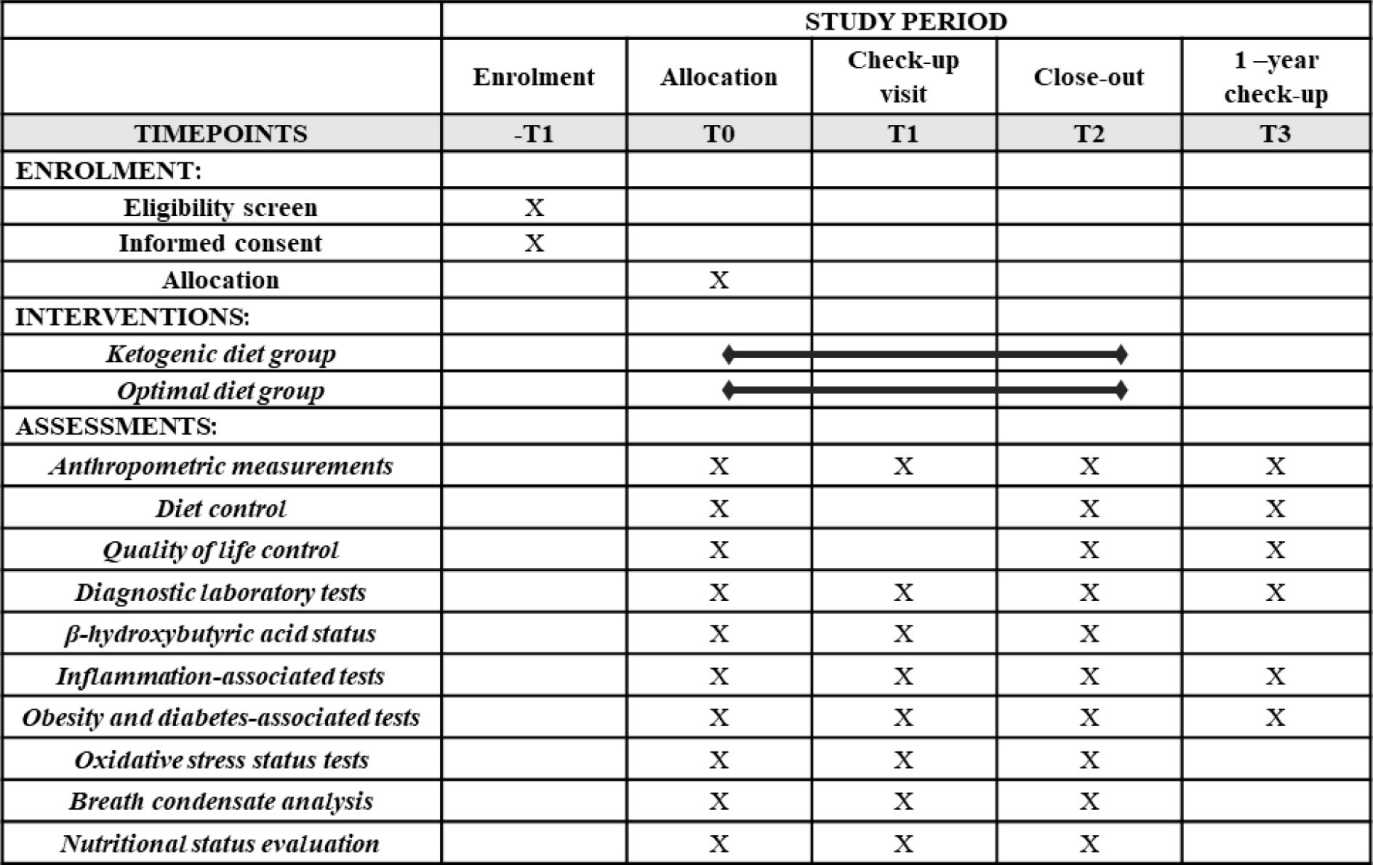
Content for the schedule of enrolment, interventions, and assessments according to SPIRIT requirements.

In the collected samples, the following laboratory analyses will be performed to verify research hypotheses, according to the SPIRIT schedule (Fig 1):

1. The analysis of β-hydroxybutyric acid in serum samples (T0-T2) using commercial colourimetric assay will inform about the ketosis state in the body and adherence to the diet in the KD group.
2. The analysis of lipid profile (total cholesterol, cholesterol HDL and LDL, triglycerides), blood morphology, and blood biochemical parameters, including liver enzymes (aspartate transglutaminase (AST), alanine transaminase (ALT), gamma-glutamyltransferase (GGT)), creatinine, uric acid, fasting glucose, insulin, haemoglobin A1c (HbA1c), C-reactive protein (CRP), albumin, total protein, ions (calcium, sodium, magnesium, potassium, phosphorus, chlorides, iron) will be performed by the Diagnostic Laboratory in Olsztyn (T0-T3) to inform about the intervention’s safety and the participants’ general body condition before, during, and after the intervention.
3. The analysis of cytokines in serum (T0-T3) using Bio-Plex Pro™ Human Cytokine 17-plex Assay (BIO-RAD, Hercules, USA), which allows for the simultaneous analysis of Granulocyte Colony-Stimulating Factor (G-CSF), Granulocyte-Macrophage Colony-Stimulating Factor (GM-CSF), interferon-gamma (IFN-γ), Monocyte chemoattractant protein-1 (MCP-1/CCL2), Macrophage Inflammatory Protein beta (MIP-β), tumour necrosis factor-alpha (TNF-α) and interleukins: IL-1β, IL-2, IL-4, IL-5, IL-6, IL-7, IL-8, IL-10, IL-12 (p70), IL-13, IL-17A as well as the analysis of inflammation-associated parameters (lipopolysaccharide-binding protein, high sensitive-CRP) analysed using commercial ELISA kits, which will inform about the inflammatory state of the study participants.
4. The analysis of obesity and diabetes markers in serum samples (T0-T3) using Bio-Plex Pro Human Diabetes 10-Plex Assay, which allows for the simultaneous analysis of C-peptide, ghrelin, gastric inhibitory peptide (GIP), glucagon-like peptide 1 (GLP-1), glucagon, insulin, leptin, plasminogen activation inhibitor-1 (PAI-1), resistin and visfatin. Moreover, adipsin and adiponectin concentrations in serum will be evaluated using Bio-Plex Pro Human Diabetes Adipsin and Adiponectin Assays. All these parameters will inform about the changes in the overweight-linked and metabolic functions.
5. The analysis of Ox markers (T0-T2), including malonaldehyde, superoxide dismutase, 8-isoprostane and 8-hydroxydeoxyguanosine using ELISA kits will inform about the effect of the diet and/or loss of body weight on the Ox in the study participants.
6. The analysis of volatile organic compounds in breath condensates (T0-T2) using solid-phase microextraction (SPME) and comprehensive two-dimensional gas chromatography with time of flight mass spectrometry (GC×GC-ToFMS) detection in breath, which will inform about the metabolic state of the study participants.
7. The analysis of amino acids in serum samples (T0-T2) using GC-MS [48], which will inform about the nutritional state and metabolic status of the study participants.
8. The analysis of fat-soluble vitamins (A, D, E & K) in serum samples (T0-T2) using high-performance liquid chromatography and ELISA kits as described previously [49, 50], which will inform about the nutritional state of the study participants.

### Urine analysis

The morning urine samples will be delivered by participants. The analysis of urine including basic urine evaluation, creatinine, uric acid, and ions (Ca, Na, Mg, K, P, Cl) will be performed by the Diagnostic Laboratory in Olsztyn (T0-T3). The analysis of the amino acid profile in urine (T0-T2) will be performed by the same method as in serum.

### Diet control

The diet will be provided daily to each participant to maintain homogeneity and to reduce the risk of drop-out caused by a lack of wish to prepare meals. Each participant will receive a study diary to record any possible side effects. All received and consumed meals from the catering and, if any, intake of the products not from the catering will have to be marked in the diary to evaluate diet compliance.

To evaluate the diet followed by the participants before and after the nutritional intervention, food frequency questionnaire-6 (FFQ-6) [51] will be applied for a retrospective semi-qualitative food intake evaluation at T0 and T3. The FFQ is well-validated and recognised as universal and independent of the geographic and subjective tools for researching eating patterns. It is also the most commonly used instrument to assess past dietary intake in epidemiological studies on the relationship between dietary factors and diseases, primarily because of its low cost and ability to capture usual dietary patterns [52]. Frequency data can explain much of the variation in dietary intake, and FFQs can provide sufficient accuracy to rank individuals in terms of risks for subsequent health outcomes. FFQs have been used in many studies to predict associations between dietary intake and disease-specific mortality and morbidity [53, 54]. The FFQ Food Consumption Frequency Questionnaire is a qualitative questionnaire, but it is also a good tool for semi-qualitative research when combined with a diary. It is a tool for collecting information on the frequency of consuming 62 assortment groups of products, representing 8 main groups of food consumed in the last 12 months. The respondents can choose from (6–10) categories of food consumption frequency: from minimal (1) never or almost never to maximal (6/10) several times per day.

### Quality of life

At the T0, T2 and T3 visits, Quality of Life (QoL) questionnaires will be completed by participants to evaluate the effect of diet and/or weight loss on the quality of life. The health-related quality of life questionnaire (HRQoL) is a suitable tool for randomised controlled trials as well as observational studies. HRQoL was introduced by CDC in 2000 [55]. The HRQoL combines three separate modules to assess perceptions of HRQoL. It is widely used by health professionals and was designed to bridge the gap between disciplines, such as sociology, psychology, and economics, about the drivers of QoL. It is for this reason that the questionnaire is fairly broad in its focus. It is available in text or graphic versions. The scale of responses ranged from 1 (excellent) to 5 (poor).

### Statistical analysis plan

The data from this two-arm parallel randomised controlled trial is amenable to analysis using standard statistical techniques. These analyses will be undertaken using STATISTICA v. 13.3 (StatSoft, Tulsa, USA) and IBM SPSS version 28.

The primary analysis for each outcome will be by intention-to-treat, meaning that all eligible consenting participants on whom an outcome is available will be included in the analysis. All statistical tests will be two-sided. A nominal significance level of alpha = 0.05 will be used to judge significance. Where appropriate, statistical significance after controlling for the False Discovery Rate will be additionally undertaken using the Benjamini-Hochberg procedure.

For each analysis, the following summaries will be provided:

- The number of participants who are included in the analysis
- A relevant summary measure of the outcome after an appropriate transformation (e.g., mean (SD), median (IQR) or number (%))
- Statistical analyses will report the effect size, 95% confidence interval and a p-value. P-values will be reported to three decimal places except where p < .001.

Analyses will be supplemented with appropriate graphical displays.

The primary outcome, weight at T2, will be compared between groups using analysis of covariance controlling for weight at T0. In general, parametric tests will be used with scale outcome data. If, for any particular analysis, there are doubts to the assuredness of validity of a parametric test then an analogous non-parametric test, will be used. ANCOVA and the non-paramteric Quade test will be used to compare randomised arms after controlling for commensurate baseline data. Simple differences between groups will be analysed using the independent sample t-test or the nonparametric Mann-Whitney test. Chemometric analyses will additionally use Principal Components Analysis (PCA), Partial Least Squares regression (PLSR) and Mutlivariable Discriminant Analysis (MDA) will be performed. The results of volatile organic compounds will be analysed using the XCMS-online platform for metabolomics. All the obtained results will be numerical and conducted in triplicates or duplicates, as appropriate.

### Data management plan

To protect the participants’ privacy and to maintain confidentiality, all personal data will be stored in password-protected files and secured against unauthorized access by third parties. The raw data and materials are only accessible to project team members. Each participant will be assigned a randomly generated code that does not allow any conclusions to be drawn about the person. Only this code will be used for naming files and samples. Only completely anonymized data will be made available to other researchers after the completion of the study or in data repositories. Only mean values and group statistics will be reported in publications.

## Discussion

We designed this prospective, single-centre, randomised controlled study to evaluate the efficacy and the effect of KD on health-related parameters in women with overweight and obesity. Obesity is a common, serious, and costly chronic disease of adults and children, which requires intervention to reduce the risk of the development of chronic diseases.

The KD is a dietetic regime with a limited intake of carbohydrates, which consists mainly of the consumption of fat which leads to a ketosis state in the body. Although originally, KD was designed for the treatment of epilepsy [56], there has been over the past few years, a societal increasing trend in following KD for body weight reduction. There is some evidence that a KD can result in a better dynamic of body weight loss at the beginning of the intervention [19]. In general, the results of studies with very low-calorie KD have reported KD to have benefit in losing weight and decreasing BMI and can confer positive effects on the human body [28, 57]. However, there is a lack of studies with a calorie-controlled control group. To investigate this knowledge gap we have described a study protocol of a comprehensive experiment that investigates the effects of KD as a weight management diet in women with overweight and obesity, with particular emphasis on inflammation, oxidative stress, nutritional status and metabolism.

### Strengths of the planned study

To date, the majority of studies with KD were performed with patients qualified for bariatric surgery, who had a very stringent caloric regime (up to 700 kcal/day) or were based on the cooking recipes given to participants. A major strength of this study is that the participants are not a clinical population, but a general population struggling with overweight and type I obesity. Moreover, in the intervention, free catering will be provided to each participant daily for the whole time of intervention, which will secure the homogeneity of the diet. Another major strength is that it is a prospective study with a comparable control group. The proposed trial is planned to recruit a sufficient number of participants to meet the sample size allowing for statistically significant results and will enjoy a sample size exceeding those used in other, albeit clinical, studies. The study participants will be women only, therefore gender as a confounding factor will be eliminated and better homogeneity of results can be obtained. In the collected samples, a wide range of analyses will be performed which will help us to understand better the physiological effects of KD. Another strength of this study is the evaluation of the long-term effects of a weight management program with KD during a follow-up visit after a year of the end of the intervention.

### Potential risks

The potential risk can be the collection of a too-low volume of blood and breath condensate. To overcome this risk, qualified and experienced nurses will be involved in the sample collection. Another risk is the problem with recruitment. To encourage participants to take part in this study, the local media will be involved to the extent allowing fulfilling the sample size. The recruitment will be initiated sufficiently earlier to collect 80 participants. The potential problem is the drop-out in the long-term data. To overcome this issue, in case of moving participants to other cities or countries, different contact data (e-mail address, phone numbers) will be collected to arrange the follow-up meeting at a suitable time for the participant.

### Limitations

Our study is limited to only women of reproductive age from a single geographical region. Other age groups, such as adolescence, women with menopause and post-menopause would be interesting targets for future follow-up trials. It would be also interesting to check if the effects observed in women will be similar in men.

### Implications

Despite above mentioned limitations, we believe that the proposed trial will contribute to a deeper understanding of how the composition of the diet is important in weight management and what physiological and metabolic effects are observed after KD.

## Conclusion

In this article, we presented a rationale and study protocol for a single-centre, two-arm nutritional intervention randomized controlled trial, to better understand the physiological and metabolic effects of KD in women with overweight and obesity.

## Supporting information

S1 Checklist SPIRIT 2013 checklist: Recommended items to address in a clinical trial protocol and related documents. (DOC)

S1 File (DOC)

S2 File (DOC)
