# Supplementary material for: The effect of isocaloric, energy-restrictive, KETOgenic diet on metabolism, inflammation, nutrition deficiencies and oxidative stress in women with overweight and obesity (KETO-MINOX): Study protocol
Source: PLoS One. 2023 May 8;18(5):e0285283. doi: 10.1371/journal.pone.0285283 (PMC10166534; doi:10.1371/journal.pone.0285283)
Supplement: S1 File — (DOC) [file pone.0285283.s002.doc]

**załącznik Nr 2**

**do Regulaminu Komisji Bioetycznej**

**Wniosek do Komisji Bioetycznej
przy Wydziale Lekarskim Collegium Medicum
Uniwersytetu Warmińsko-Mazurskiego w Olsztynie**

**pt.** **Ocena wpływu izokalorycznej, redukcyjnej diety ketogenicznej na metabolizm, stan zapalny, wybrane parametry odżywienia oraz stres oksydacyjny kobiet z nadwagą i otyłością**

**WNIOSEK DO KOMISJI BIOETYCZNEJ O WYDANIE OPINII**

- o badaniu klinicznym ⁯

- o eksperymencie medycznym ⁯

- o eksperymencie leczniczym ⁯

- o eksperymencie badawczym ⁯

- o badaniu naukowym X

1. **DANE IDENTYFIKACYJNE WNIOSKODAWCY**

Nazwa instytucji, wydziału, katedry, kliniki (adres jednostki prowadzącej badanie)

Instytut Rozrodu Zwierząt i Badań Żywności Polskiej Akademii Nauk w Olsztynie, Zakład Chemii i Biodynamiki Żywności, ul. Tuwima 10, 10-748 Olsztyn

Uniwersytet Warmińsko-Mazurski w Olsztynie, Wydział Lekarski, Collegium Medicum, Katedra Medycyny Rodzinnej i Chorób Zakaźnych, ul. Warszawska 30, 10-082 Olsztyn

**Główny Badacz**

Imię i nazwisko: Natalia Drabińska

Stopień/tytuł naukowy, specjalizacja: Doktor. Dziedzina wiodąca: nauk rolniczych/technologia żywności i żywienia, Dziedzina i dyscyplina dodatkowa: nauk medycznych i nauk o zdrowiu/nauki o zdrowiu

Numer telefonu: +48 733 814 404

Adres e-mail: n.drabinska@pan.olsztyn.pl

1. **AKTUALNY STAN WIEDZY BĘDĄCY PRZEDMIOTEM BADANIA
   (z załączonym piśmiennictwem)**

Otyłość jest uważana za jeden z największych problemów XXI wieku. Jest definiowana jako nieprawidłowa lub nadmierna akumulacja tkanki tłuszczowej, która zwiększa ryzyko zdrowotne. Rodzaj otyłości określa się na podstawie wskaźnika masy ciała (BMI), liczonego jako masa ciała (kg) podzielona przez kwadrat wzrostu (m^2^), gdzie BMI 25 kg/m^2^ i więcej jest klasyfikowane jako nadwaga. BMI powyżej 30 kg/m^2^ jest klasyfikowane jako otyłość, którą można podzielić na I (BMI: 30,0 – 34,9 kg/m^2^), II (BMI: 35,0 – 39,9 kg/m2) i III (BMI: powyżej 40,0 kg/m2 ) klasę otyłości (Arrone 2002). Według Światowej Organizacji Zdrowia (WHO) w 2016 r. nadwaga występowała u prawie 2 mld osób dorosłych na świecie, a około 650 mln ludzi spełnia wspomniane kryterium otyłości. W Europie szacuje się, że nawet do 50% populacji może mieć podwyższoną masę ciała (WHO 2016). Według The Global Burden of Disease Study opublikowanego w 2010 r., przypadki śmiertelne spowodowane konsekwencjami otyłości występują częściej niż spowodowane niedożywieniem i głodem (Lozano i in., 2012).

Rozpowszechnienie występowania otyłości i nadwagi wynika z popularności tzw. diety zachodniej, charakteryzującej się nadmierną konsumpcją wysoko przetworzonych produktów spożywczych. Dieta z nadwyżką kaloryczną w połączeniu z niską aktywnością fizyczną prowadzi do zachwiania równowagi energetycznej i odkładania się tłuszczu. Co ważne, organizm skuteczniej chroni organizm przed głodem niż przybieraniem na wadze, łatwiej jest więc nabrać dodatkowe kilogramy niż je stracić (Williams et al., 2015). Na rozwój otyłości w wieku dorosłym mogą wpływać czynniki środowiskowe, genetyczne i epigenetyczne już w okresie prenatalnym (Lin i in., 2017). Ponadto czynniki fizjologiczno-genetyczne związane z rozwojem otyłości, wpływają na spożycie kalorii, termogenezę, wykorzystanie lipidów i obrót składnikami odżywczymi (Lin i in., 2017). Układ hormonalny reguluje uczucie sytości i apetytu poprzez adipokiny wydzielane przez tkankę tłuszczową (Ouchi et al., 2011). Przerost tkanki tłuszczowej modyfikuje jej status wydzielniczy, prowadząc do rozwoju stanu zapalnego o niskim nasileniu.

Nadwaga to nie tylko problem estetyczny. Podwyższona masa ciała zwiększa ryzyko cukrzycy typu 2, chorób sercowo-naczyniowych, w tym nadciśnienia, oraz niektórych rodzajów nowotworów (Williams i in., 2015). Występowanie chorób związanych z otyłością jest ściśle związane z rozmieszczeniem tkanki tłuszczowej, a w szczególności z lokalizacją trzewną. Nagromadzenie trzewnej tkanki tłuszczowej wpływa na stężenie adipokin, w konsekwencji przyczynia się do rozwoju przewlekłego stanu zapalnego i zaburzeń metabolicznych (Graßmann i in., 2017). Adipokiny wydzielane przez trzewną tkankę tłuszczową odgrywające rolę w zapaleniu, metabolizmie lub rozwoju chorób układu krążenia to czynnik martwicy nowotworu alfa (TNF-a), interleukina-6 (IL-6), IL-beta, adiponektyna, leptyna, rezystyna, surowiczy amyloid A-3 (SAA3), kwaśna glikoproteina alfa-1, pentraksyna-3, antagonista receptora IL-1, czynnik hamujący migrację makrofagów, inhibitor aktywatora plazminogenu-1 (PAI-1), wisfatyna, czynnik wzrostu śródbłonka naczyniowego (VEGF) i wiele innych (Fasshauer i Blüher, 2015; Su i Peng, 2020). Adipokiny indukują również produkcję reaktywnych form tlenu (ROS), generując stres oksydacyjny (Ox) i nieregularną produkcję innych adipokin (Marseglia i in., 2014). W generowaniu Ox w otyłości zaangażowanych jest kilka mechanizmów, a Ox i procesy prozapalne są ze sobą ściśle powiązane. Po aktywacji wiele komórek odpornościowych wytwarza ROS i promuje stan zapalny (Marseglia i in., 2014). Nadmierna akumulacja tłuszczu u pacjentów z otyłością prowadzi do patologicznego wzrostu poziomu wolnych kwasów tłuszczowych w surowicy, zaburza metabolizm glukozy, sprzyja akumulacji substratów energetycznych w wątrobie, mięśniach i tkance tłuszczowej (tłuszcze i glukoza) oraz sprzyja podwyższeniu poziomu mitochondriów i utlenianiu peroksysomów. W konsekwencji prowadzi do syntezy wolnych rodników, Ox, uszkodzenia mitochondrialnego DNA i wreszcie do lipotoksyczności, obejmującej różne niekorzystne oddziaływania kwasów tłuszczowych na struktury komórkowe (Goossens, 2008). Uszkodzenie komórek prowadzi do wysokiej produkcji cytokin, takich jak TNF-α, który generuje dalsze ROS w tkankach i zwiększa szybkość peroksydacji lipidów.

Choroby współistniejące z otyłością, lub przez nią indukowane, takie jak zespół metaboliczny, problemy z oddychaniem i upośledzenie funkcji poznawczych, mają niekorzystny wpływ na śmiertelność ogólną oraz przedwczesną (González-Muniesa i in., 2017; Jauch-Chara i Oltmanns, 2014; Franks i in. 2010).

Ograniczenie kalorii to najczęściej stosowany sposób na odchudzanie. Ogólnie rzecz biorąc, nie ma jednej najlepszej strategii żywieniowej, jeśli chodzi o przestrzeganie diety i utrzymywanie restrykcji energetycznych (Anton i in., 2017; Johnston i in., 2014). Kluczową kwestią jest, nie tylko jaki rodzaj diety wybrać, aby skutecznie stracić nadmiar masy ciała, ale też aby uniknąć efektu „jojo” co będzie kontynuowane po okresie redukcji. Wreszcie, dieta ma być bezpieczna, łatwa w przestrzeganiu i nie powodująca negatywnych skutków. Ujemny bilans energetyczny ma znaczenie kluczowe dla powodzenia diety (Abete i in., 2010). Przeprowadzono wiele żywieniowych badań klinicznych pod kątem fizjologicznych skutków leczenia otyłości skoncentrowanych na ocenie różnych rodzajów diety, w tym niskokalorycznych (Luglio i in., 2017), bardzo niskokalorycznych (Umphonsathien i in., 2019), wysokobiałkowych (Benedí i in., 2017), diety niskotłuszczowej (Biolato i in., 2019) oraz postu przerywanego (Liu i in., 2019). Kolejną dietą, do tej pory z powodzeniem stosowaną jako terapia wspomagająca w leczeniu padaczki (Ułamek-Kozioł i in., 2019) jest dieta ketogeniczna (KD). Dieta ta obecnie cieszy się rosnącym zainteresowaniu jako metoda redukcji masy ciała. Według badania przeprowadzonego przez Fundację Międzynarodowej Rady Informacji o Żywności (IFIC) z udziałem ponad 1000 Amerykanów, KD jest jedyną dietą o wzrastającym zainteresowaniu (lata 2018-2019), u osób których motywacją była utrata wagi (https://foodinsight.org). Mimo, że popularność tej diety rośnie w społeczeństwie, wiedza na temat bezpieczeństwa i skuteczności KD w odchudzaniu jest ograniczona.

Termin KD charakteryzuje dietę wysokotłuszczową i nisko węglowodanową skutkującą stanem ketozy w organizmie. Ideą KD było naśladowanie fizjologicznego stanu głodu bez stosowania postu. Warto podkreślić, że nie każda dieta nisko węglowodanowa jest ketogeniczna. W diecie nisko węglowodanowej z wysokim spożyciem białka nie obserwuje się ketozy. Wynika to z faktu, że wiele z aminokwasów jest przekształcane do glukozy, co zapobiega występowaniu ketozy (VanItallie & Nufert, 2003).

Wcześniejsze badania z zastosowaniem KD w otyłości wykazały istotne zmniejszenie masy ciała, BMI i zawartości tkanki tłuszczowej w organizmie po stosowaniu KD. Nie potwierdzono jednak, że wynika to ze stanu ketozy, ale z bardzo ograniczonego spożycia kalorii. W większości tych badań nad zastosowaniem KD w leczeniu otyłości zastosowano bardzo niskokaloryczne diety, ograniczone do zaledwie 500-800 kcal/dzień. Można podejrzewać, że obserwowane zmiany wynikały ze znacznego ograniczonego spożycia, a nie ze składu odżywczego diety, zwłaszcza, że ​​większość badań nie obejmowała grup kontrolnych do porównania wpływu różnych składów odżywczych diet (Castro i in. , 2018; Hall i wsp., 2016; Schiavo i wsp., 2018).

Jeśli chodzi o efekty metaboliczne, bardzo niskokaloryczna KD spowodowała wyraźne zmiany w poziomach krążących miokin, w tym IL-6, IL-8 i metaloproteinazy 2, co sugeruje korzystne aspekty zdrowotne (Sajoux i in., 2019). Ponadto, niskokaloryczna KD uzupełniona w DHA obniżała stężenie insuliny, triglicerydów, cholesterolu całkowitego, cholesterolu LDL, białka C-reaktywnego, TNF-α i rezystyny ​​(de Luis i in., 2016). Pozytywny wpływ bardzo niskokalorycznej KD na metabolizm lipidów i parametry związane z cukrzycą potwierdzili również inni autorzy (Castaldo i in., 2016; Goday i in., 2016; Ministrini i in., 2019; Pilone i in. , 2018). Dodatkowo bardzo niskokaloryczna KD skutkowała obniżeniem ciśnienia tętniczego krwi (Castaldo i in., 2016; Cicero i in., 2015). Jednak wszystkie te efekty uzyskano w badaniach z bardzo niskokaloryczną KD, dedykowaną osobom zakwalifikowanym do operacji bariatrycznej i obserwowanych pod nadzorem klinicznym. Wyników tych badań nie można porównać z efektem KD z odpowiednią podażą kalorii lub jedynie jej nieznaczną redukcją, którą stosują osoby zmotywowane do odchudzania i zainspirowane przez media społecznościowe i influencerów do stosowania KD.

Opisano niewiele badań dotyczących KD z optymalnym spożyciem kalorii (Hall i in., 2016; Kenig i in., 2019; Mohorko i in., 2019a; Schiavo i in., 2018). Większość z tych badań nie obejmowała grupy kontrolnej, więc wyniki są trudne do interpretacji. Biorąc pod uwagę badania nad KD, które obejmowały grupę kontrolną, pozytywny wpływ KD na leczenie otyłości potwierdzono w badaniu z udziałem 45 otyłych kobiet z nowotworem jajnika lub endometrium (Cohen i in., 2018). Po dwunastu tygodniach autorzy zaobserwowali znaczącą utratę trzewnej, androidalnej i całkowitej masy tłuszczowej u osób po KD w porównaniu do grupy kontrolnej. KD była również związana z niższym poziomem insuliny w surowicy na czczo u otyłych kobiet z rakiem jajnika lub endometrium, jak sugerują autorzy, zwiększając wrażliwość na insulinę (Cohen i in., 2018). Większość badań z zastosowaniem KD przy optymalnym spożyciu kalorii skupiała się wyłącznie na zmianach masy i składu ciała oraz wpływie na parametry kardiologiczne (Drabińska i in., 2021). Mając powyższe na uwadze istnieje potrzeba kompleksowych badań nad fizjologicznym wpływem KD u osób z nadwagą, zwłaszcza w zakresie obecności stanu zapalnego, odżywiania i zmian metabolicznych.

Z drugiej strony sugerowano, że długotrwałe stosowanie KD wiąże się z ryzykiem wystąpienia kamicy nerkowej, zwiększonego poziomu kwasu moczowego we krwi i osteoporozy (niskie spożycia wapnia). Nalezy jednak podkreślić, że długotrwałą KD stosują głównie pacjenci z padaczką lekooporną w celu zmniejszenia częstości występowania napadów (Ułamek-Kozioł i in., 2019). Dieta u osób z otyłością i nadwagą jest stosowana w celu obniżenia wagi, stąd stosowane są krótkie interwencje dietetyczne, zwykle nie trwające dłużej niż kilka tygodni. Skutki uboczne takich interwencji są stosunkowo łagodne i obejmują głód, zmęczenie, obniżony nastrój, drażliwość, zaparcia i bóle głowy.

Interesującym zagadnieniem jest też sprawdzenie, czy skład odżywczy diety wpływa na masę ciała po okresie obserwacji, nawet bez ścisłej kontroli diety w międzyczasie.

Podsumowując, dostępna literatura naukowa wskazuje, że stosowanie KD może być korzystne w leczeniu otyłości, jednak nadal brakuje kompleksowych badań, które potwierdzają skuteczność i bezpieczeństwo stosowania KD w celu utraty masy ciała. Do tej pory większość badań miała małą liczebność próby, brak grup kontrolnych a interwencja była krótkoterminowa. Co więcej, większość badań z KD przeprowadzono z bardzo niskokaloryczną KD, a korzystny wpływ na masę ciała można było tu wiązać z deficytem kalorycznym, a nie ze składem odżywczym stosowanej diety. Obecność tylko nielicznych badań przeprowadzonych nad niedoborami żywieniowymi (Kenig i in., 2019), sugeruje potrzebę weryfikacji danych dotyczących spożycia minerałów, witamin i składników odżywczych oraz ich stężenia w organizmie.

Biorąc pod uwagę wszystkie przedstawione powyżej informacje, konieczna i uzasadniona wydaje się być intensywna i kompleksowa analiza efektów fizjologicznych KD w sposób randomizowany i kontrolowany. Projekt ma na celu lepsze zrozumienie efektów metabolicznych KD u osób z nadwagą w sposób kontrolowany, typowy dla badań żywieniowych – catering żywnościowy zbilansowany przez doświadczonego dietetyka, dostarczany codziennie każdemu uczestnikowi. Według wiedzy autorów nie ma zakończonych ani trwających badań skoncentrowanych na skuteczności KD w układzie randomizowanym, z odpowiednio zbilansowaną grupą kontrolną, z jednorodną dietą podawaną wszystkim uczestnikom podczas całej interwencji i analizowaniem wielu parametrów jednocześnie w jednym badaniu. W Projekcie zostaną wykorzystane innowacyjne i wydajne techniki i testy do pomiaru maksymalnej liczby parametrów z jak najmniejszej ilości materiału biologicznego i w jak najkrótszym czasie. Wyniki projektu bezpośrednio odpowiedzą na pytania zadawane przez wiele osób zmagających się z otyłością i nadwagą, które zastanawiają się, czy KD jest bezpiecznym i skutecznym sposobem na odchudzanie. Projekt wniesie istotny wkład w naukę o żywności, dietetykę, żywienie człowieka i metabolomikę.

**Referencje:**

1. Abete, I., Astrup, A., Martínez, J. A., Thorsdottir, I., & Zulet, M. A. (2010). Obesity and the metabolic syndrome: Role of different dietary macronutrient distribution patterns and specific nutritional components on weight loss and maintenance. Nutrition Reviews, 68(4), 214–231. https://doi.org/10.1111/j.1753-4887.2010.00280.x
2. Anton, S. D., Hida, A., Heekin, K., Sowalsky, K., Karabetian, C., Mutchie, H., Leeuwenburgh, C., Manini, T. M., & Barnett, T. E. (2017). Effects of Popular Diets without Specific Calorie Targets on Weight Loss Outcomes: Systematic Review of Findings from Clinical Trials. Nutrients, 9(8), 822. https://doi.org/10.3390/nu9080822
3. Aronne, L. J. (2002). Classification of Obesity and Assessment of Obesity-Related Health Risks. Obesity Research, 10(S12), 105S-115S. https://doi.org/10.1038/oby.2002.203
4. Benedí, M. V. M., Calahorra, S. P., Sanz, A. M. B., Baila Rueda, L., Lamiquiz Moneo, I., Cenarro, A., Civeira, F., & Gallego, R. M. (2017). A randomized, open-label study to investigate the effect of a high protein diet compared to a normoprotein diet on hydrocarbon metabolism in patients with diabetes or prediabetes and obesity. Atherosclerosis, 263, e263. https://doi.org/10.1016/j.atherosclerosis.2017.06.851
5. Biolato, M., Manca, F., Marrone, G., Cefalo, C., Racco, S., Miggiano, G. A., Valenza, V., Gasbarrini, A., Miele, L., & Grieco, A. (2019). Intestinal permeability after Mediterranean diet and low-fat diet in non-alcoholic fatty liver disease. World Journal of Gastroenterology, 25(4), 509–520. https://doi.org/10.3748/wjg.v25.i4.509
6. Castaldo, G., Monaco, L., Castaldo, L., Galdo, G., & Cereda, E. (2016). An observational study of sequential protein-sparing, very low-calorie ketogenic diet (Oloproteic diet) and hypocaloric Mediterranean-like diet for the treatment of obesity. International Journal of Food Sciences and Nutrition, 67(6), 696–706. https://doi.org/10.1080/09637486.2016.1186157
7. Castro, A. I., Gomez-Arbelaez, D., Crujeiras, A. B., Granero, R., Aguera, Z., Jimenez-Murcia, S., Sajoux, I., Lopez-Jaramillo, P., Fernandez-Aranda, F., & Casanueva, F. F. (2018). Effect of a very low-calorie ketogenic diet on food and alcohol cravings, physical and sexual activity, sleep disturbances, and quality of life in obese patients. Nutrients, 10(10). https://doi.org/10.3390/nu10101348
8. Cicero, A. F. G., Benelli, M., Brancaleoni, M., Dainelli, G., Merlini, D., & Negri, R. (2015). Middle and Long-Term Impact of a Very Low-Carbohydrate Ketogenic Diet on Cardiometabolic Factors: A Multi-Center, Cross-Sectional, Clinical Study. High Blood Pressure and Cardiovascular Prevention, 22(4), 389–394. https://doi.org/10.1007/s40292-015-0096-1
9. Cohen, C. W., Fontaine, K. R., Arend, R. C., Alvarez, R. D., Leath, C. A., Huh, W. K., Bevis, K. S., Kim, K. H., Straughn, J. M., & Gower, B. A. (2018). A ketogenic diet reduces central obesity and serum insulin in women with ovarian or endometrial cancer. Journal of Nutrition, 148(8), 1253–1260. https://doi.org/10.1093/jn/nxy119
10. de Luis, D., Domingo, J. C., Izaola, O., Casanueva, F. F., Bellido, D., & Sajoux, I. (2016). Effect of DHA supplementation in a very low-calorie ketogenic diet in the treatment of obesity: a randomized clinical trial. Endocrine, 54, 111–122. https://doi.org/10.1007/s12020-016-0964-z
11. Drabińska, N., Wiczkowski, W., & Piskuła, M. K. (2021). Recent advances in the application of a ketogenic diet for obesity management. Trends in Food Science and Technology, 110. https://doi.org/10.1016/j.tifs.2021.01.080
12. Fasshauer, M., & Blüher, M. (2015). Adipokines in health and disease. Trends in Pharmacological Sciences, 36(7), 461–470. https://doi.org/10.1016/j.tips.2015.04.014
13. Franks, P. W., Hanson, R. L., Knowler, W. C., Sievers, M. L., Bennett, P. H., & Looker, H. C. (2010). Childhood obesity, other cardiovascular risk factors, and premature death. New England Journal of Medicine, 362(6), 485-493.
14. Goday, A., Bellido, D., Sajoux, I., Crujeiras, A. B., Burguera, B., García-Luna, P. P., Oleaga, A., Moreno, B., & Casanueva, F. F. (2016). Short-Term safety, tolerability and efficacy of a very low-calorie-ketogenic diet interventional weight loss program versus hypocaloric diet in patients with type 2 diabetes mellitus. Nutrition and Diabetes, 6(9), e230. https://doi.org/10.1038/nutd.2016.36
15. González-Muniesa, P., Mártinez-González, M.-A., Hu, F. B., Després, J.-P., Matsuzawa, Y., F Loos, R. J., Moreno, L. A., Bray, G. A., & Alfredo Martinez, J. (2017). Obesity. Nature Reviews, 3, 17034. https://doi.org/10.1038/nrdp.2017.34
16. Goossens, G. H. (2008). The role of adipose tissue dysfunction in the pathogenesis of obesity-related insulin resistance. Physiology & Behavior, 94(2), 206–218. https://doi.org/https://doi.org/10.1016/j.physbeh.2007.10.010
17. Graßmann, S., Wirsching, J., Eichelmann, F., & Aleksandrova, K. (2017). Association Between Peripheral Adipokines and Inflammation Markers: A Systematic Review and Meta-Analysis. Obesity, 25(10), 1776–1785. https://doi.org/10.1002/oby.21945
18. Hall, K. D., Chen, K. Y., Guo, J., Lam, Y., Liebel, R. L., Mayer, L., Reitman, M. L., Rosenbaum, M., Smith, S. R., Walsh, B. T., & Ravussin, E. (2016). Energy expenditure and body composition changes after an isocaloric ketogenic diet in overweight and obese men. American Journal of Clinical Nutrition, 104(2), 324–333. https://doi.org/10.1111/obr.12399
19. Jauch-Chara, K., & Oltmanns, K. M. (2014). Obesity - A neuropsychological disease? Systematic review and neuropsychological model. Progress in Neurobiology, 114, 84–101. https://doi.org/10.1016/j.pneurobio.2013.12.001
20. Johnston, B. C., Kanters, S., Bandayrel, K., Wu, P., Naji, F., Siemieniuk, R. A., Ball, G. D. C., Busse, J. W., Thorlund, K., Guyatt, G., Jansen, J. P., & Mills, E. J. (2014). Comparison of Weight Loss Among Named Diet Programs in Overweight and Obese Adults: A Meta-analysis. JAMA, 312(9), 923–933. https://doi.org/10.1001/jama.2014.10397
21. Kenig, S., Petelin, A., Poklar Vatovec, T., Mohorko, N., & Jenko-Pražnikar, Z. (2019). Assessment of micronutrients in a 12-wk ketogenic diet in obese adults. Nutrition, 67–68, 2–8. https://doi.org/10.1016/j.nut.2019.06.003
22. Lin, X., Lim, I. Y., Wu, Y., Teh, A. L., Chen, L., Aris, I. M., Soh, S. E., Tint, M. T., MacIsaac, J. L., Morin, A. M., Yap, F., Tan, K. H., Saw, S. M., Kobor, M. S., Meaney, M. J., Godfrey, K. M., Chong, Y. S., Holbrook, J. D., Lee, Y. S., … group, G. study. (2017). Developmental pathways to adiposity begin before birth and are influenced by genotype, prenatal environment and epigenome. BMC Medicine, 15(1), 50. https://doi.org/10.1186/s12916-017-0800-1
23. Liu, B., Hutchison, A. T., Thompson, C. H., Lange, K., & Heilbronn, L. K. (2019). Markers of adipose tissue inflammation are transiently elevated during intermittent fasting in women who are overweight or obese. Obesity Research and Clinical Practice, 13(4), 408–415. https://doi.org/10.1016/j.orcp.2019.07.001
24. Lozano, R., Naghavi, M., Foreman, K., Lim, S., Shibuya, K., Aboyans, V., Abraham, J., Adair, T., Aggarwal, R., Ahn, S. Y., AlMazroa, M. A., Alvarado, M., Anderson, H. R., Anderson, L. M., Andrews, K. G., Atkinson, C., Baddour, L. M., Barker-Collo, S., Bartels, D. H., … Murray, C. J. L. (2012). Global and regional mortality from 235 causes of death for 20 age groups in 1990 and 2010: A systematic analysis for the Global Burden of Disease Study 2010. The Lancet, 380, 2095–2128. https://doi.org/10.1016/S0140-6736(12)61728-0
25. Luglio, H. F., Sulistyoningrum, D. C., Muharomin, I. R., & Huriyati, E. (2017). Leptin, appetite and weight rebound in overweight/obesity individuals undertook weight loss program using a low calorie diet with or without exercise. Mediterranean Journal of Nutrition and Metabolism, 10(3), 223–233. https://doi.org/10.3233/mnm-17162
26. Marseglia, L., Manti, S., D’Angelo, G., Nicotera, A., Parisi, E., Di Rosa, G., Gitto, E., & Arrigo, T. (2014). Oxidative stress in obesity: a critical component in human diseases. International Journal of Molecular Sciences, 16(1), 378–400. https://doi.org/10.3390/ijms16010378
27. Ministrini, S., Calzini, L., Nulli Migliola, E., Ricci, M. A., Roscini, A. R., Siepi, D., Tozzi, G., Daviddi, G., Martorelli, E.-E., Paganelli, M. T., & Lupattelli, G. (2019). Lysosomal Acid Lipase as a Molecular Target of the Very Low Carbohydrate Ketogenic Diet in Morbidly Obese Patients: The Potential Effects on Liver Steatosis and Cardiovascular Risk Factors. Journal of Clinical Medicine, 8, 621. https://doi.org/10.3390/jcm8050621
28. Mohorko, N., Černelič-Bizjak, M., Poklar-Vatovec, T., Grom, G., Kenig, S., Petelin, A., & Jenko-Pražnikar, Z. (2019a). Weight loss, improved physical performance, cognitive function, eating behavior, and metabolic profile in a 12-week ketogenic diet in obese adults. Nutrition Research, 62, 64–77. https://doi.org/10.1016/j.nutres.2018.11.007
29. Mohorko, N., Černelič-Bizjak, M., Poklar-Vatovec, T., Grom, G., Kenig, S., Petelin, A., & Jenko-Pražnikar, Z. (2019b). Weight loss, improved physical performance, cognitive function, eating behavior, and metabolic profile in a 12-week ketogenic diet in obese adults. Nutrition Research, 62, 64–77. https://doi.org/10.1016/j.nutres.2018.11.007
30. Ouchi, N., Parker, J. L., Lugus, J. J., & Walsh, K. (2011). Adipokines in inflammation and metabolic disease. Nature Reviews Immunology, 11(2), 85–97. https://doi.org/10.1038/nri2921
31. Pilone, V., Tramontano, S., Renzulli, M., Romano, M., Cobellis, L., Berselli, T., & Schiavo, L. (2018). Metabolic effects, safety, and acceptability of very low-calorie ketogenic dietetic scheme on candidates for bariatric surgery. Surgery for Obesity and Related Diseases, 14(7), 1013–1019. https://doi.org/10.1016/j.soard.2018.03.018
32. Sajoux, I., Lorenzo, P. M., Gomez-Arbelaez, D., Zulet, M. A., Abete, I., Castro, A. I., Baltar, J., Portillo, M. P., Tinahones, F. J., Martinez, J. A., Crujeiras, A. B., & Casanueva, F. F. (2019). Effect of a Very-Low-Calorie Ketogenic Diet on Circulating Myokine Levels Compared with the Effect of Bariatric Surgery or a Low-Calorie Diet in Patients with Obesity. Nutrients, 11(10). https://doi.org/10.3390/nu11102368
33. Schiavo, L., Pilone, V., Rossetti, G., Barbarisi, A., Cesaretti, M., & Iannelli, A. (2018). A 4-Week Preoperative Ketogenic Micronutrient-Enriched Diet Is Effective in Reducing Body Weight, Left Hepatic Lobe Volume, and Micronutrient Deficiencies in Patients Undergoing Bariatric Surgery: a Prospective Pilot Study. Obesity Surgery, 28(8), 2215–2224. https://doi.org/10.1007/s11695-018-3145-8
34. Su, X., & Peng, D. (2020). Adipokines as novel biomarkers of cardio-metabolic disorders. Clinica Chimica Acta, 507, 31–38. https://doi.org/https://doi.org/10.1016/j.cca.2020.04.009
35. Ułamek-Kozioł, M., Czuczwar, S. J., Januszewski, S., & Pluta, R. (2019). Ketogenic Diet and Epilepsy. Nutrients, 11(10), 2510. https://doi.org/10.3390/nu11102510
36. Umphonsathien, M., Prutanopajai, P., Aiam-O-Ran, J., Thararoop, T., Karin, A., Kanjanapha, C., Jiamjarasrangsi, W., & Khovidhunkit, W. (2019). Immediate and long-term effects of a very-low-calorie diet on diabetes remission and glycemic control in obese Thai patients with type 2 diabetes mellitus. Food Science and Nutrition, 7(3), 1113–1122. https://doi.org/10.1002/fsn3.956
37. VanItallie, T. B., & Nufert, T. H. (2003). Ketones : Metabolism ’ s Ugly Duckling. Nutrition Reviews, 61(10), 327–341. https://doi.org/10.131/nr.2003.oct.327
38. WHO. (2016). Obesity and overweight: Fact sheet. In WHO Media Centre.
39. Williams, E. P., Mesidor, M., Winters, K., Dubbert, P. M., & Wyatt, S. B. (2015). Overweight and Obesity: Prevalence, Consequences, and Causes of a Growing Public Health Problem. Current Obesity Reports, 4(3), 363–370. https://doi.org/10.1007/s13679-015-0169-4
    1. Proszę wskazać schorzenie(-a) lub chorobę(-y), będące przedmiotem badania (opis słowny)

# Nadwaga i otyłość

- 1. Czy schorzenie spełnia kryteria choroby rzadko występującej? tak ⁯ nie X
  2. Czy badanie dotyczy: (proszę zaznaczyć wszystkie punkty, których dotyczy badanie)
     1. Diagnostyka ⁯
     2. Profilaktyka X
     3. Leczenie ⁯
     4. Bezpieczeństwo stosowania X
     5. Skuteczność X
     6. Farmakokinetyka ⁯
     7. Farmakodynamika ⁯
     8. Biorównoważność ⁯
     9. Zależność między dawką a odpowiedzią ⁯
     10. Farmakogenetyka ⁯
     11. Farmakogenomika ⁯
     12. Farmakoekonomika ⁯
     13. Inne ⁯

Jeżeli „inne”, proszę podać jakie:…………………………………

1. **CELE BADANIA**
   1. Cel główny: …

# Podstawowym celem jest ocena wpływu stosowania diety ketogenicznej jako diety odchudzającej na stan zapalny i stres oksydacyjny u kobiet z nadwagą i otyłością

- 1. Cele drugorzędowe:

# Cele drugorzędne to:

1) ocena wpływu diety ketogenicznej na masę i skład ciała

2) ocena wpływu utraty masy ciała związanej z dietą ketogeniczną na stan odżywienia kobiet z nadwagą i otyłością

3) ocena wpływu odchudzania związanego z dietą ketogeniczną na profil metabolitów w wydychanym powietrzu,

4) ocena wpływu odchudzania związanego z dietą ketogeniczną na parametry związane z otyłością i cukrzycą, w tym profil lipidowy, status adipokin

5) ocena długofalowych efektów i skuteczności diety ketogenicznej

6) ocena wpływu diety ketogenicznej na stan mikrobioty jelitowej

- 1. Czy występują podbadania? tak ⁯ nie X

Jeżeli „tak”, proszę podać pełny tytuł, datę i wersję każdego podbadania oraz ich cele związane
z badaniem: ………………………………………………………………………..

1. **GŁÓWNE KRYTERIA WŁĄCZENIA** (proszę podać wykaz najważniejszych):

Kobiety w wieku 18-45 lat (w okresie rozrodczym), stosowana antykoncepcja hormonalna lub mechaniczna w trakcie badania, BMI 25,5 – 35, motywacja do redukcji masy ciała oraz udziału w badaniu

**GŁÓWNE KRYTERIA WYŁĄCZENIA** (proszę podać wykaz najważniejszych):

Nadwaga/otyłość wynikająca z chorób genetycznych, choroby endokrynologiczne, autoimmunologiczne, psychiatryczne, ciąża, karmienie piersią, cukrzyca, inne chroniczne choroby wymagające farmakoterapii, udział w innych badaniach klinicznych, otyłość typu II i III (BMI > 35), spadek masy ciała > 3 kg w ciągu 12 wygodni przez rozpoczęciem badań, duże zmiany intensywności ćwiczeń fizycznych w okresie 4 tygodni przed rozpoczęciem badań

1. **GŁÓWNY(-E) PUNKT(-Y) KOŃCOWY(-E) BADANIA:**

Głównym punktem końcowym badania jest tu zmiana wagi ciała ochotniczki w dniu zakończenia diety. Ponadto ocenione zostaną: poziomy parametrów lipidowych oraz morfologia krwi, poziom cytokin oraz parametrów związanych z rozwojem otyłości i cukrzycy, parametry stresu oksydacyjnego, stężenie witamin rozpuszczalnych w tłuszczach i aminokwasów, metabolitów oddechu oraz mikrobioty jelitowej po zakończeniu diety oraz po 12 miesiącach od zakończenia diety.

1. **PLAN BADANIA**
   1. Kontrolowane tak X nie ⁯

Jeżeli „tak”, proszę zaznaczyć: …

- - 1. Randomizowane tak X nie ⁯
    2. Otwarte tak X nie ⁯
    3. Pojedynczo zaślepione tak ⁯ nie X
    4. Podwójnie zaślepione tak ⁯ nie X
    5. W grupach równoległych tak X nie ⁯
    6. Krzyżowe tak ⁯ nie X
    7. Prospektywne tak X nie ⁯
    8. Retrospektywne tak ⁯ nie X
    9. Inne tak ⁯ nie X

Jeżeli „inne”, proszę podać jakie: …

- 1. EFEKTY UBOCZNE (załącznik nr 3)
     1. Metody rejestracji efektów ubocznych

Efekty uboczne, o ile wystąpią, będą rejestrowane przez uczestników w dzienniczku badania.

- - 1. Sposoby postępowania w wypadku stwierdzenia powikłań.

Osoby biorące udział w badaniu, u których zaistnieje podejrzenie niekorzystnego wpływu diety na ich stan zdrowia zostaną poddane ocenie lekarskiej (lek. Jerzy Romaszko, lek. Sebastian Borowicz-Skoneczny), a w razie jego potwierdzenia, zostaną wyłączone z badania. Łagodne dolegliwości gastryczne wynikające ze zmiany diety nie są tu wskazaniem do wyłączenia z badania. Zachorowania wynikające z innych przyczyn będą leczone w ramach systemu opieki zdrowotnej. W zależności od ich charakteru mogą, ale nie muszą być wskazaniem do wykluczenia z udziału w badaniu. Każdy taki przypadek będzie oceniany indywidualnie.

- 1. Definicja zakończenia badania:

Badanie zostanie zakończone po 12 miesiącach od zakończenia stosowania diety. Każdy z uczestników może przerwać udział w badaniu w dowolnym momencie.

- 1. Wstępny przewidywany czas prowadzenia badania:

Przewidywany termin rozpoczęcia badania (dzień, miesiąc, rok) 1.04.2023

Przewidywany termin zakończenia badania (dzień, miesiąc, rok) 1.06.2024

- 1. zgodnie z uchwałą badacz składa sprawozdanie z wykonania badania
     w terminie określonym w uchwale.

1. **GRUPY PACJENTÓW**
   1. Grupy wiekowe
      1. Poniżej 18 roku życia tak ⁯ nie X

Jeżeli „tak”, proszę wskazać:

- - - 1. Rozwój wewnątrzmaciczny tak ⁯ nie X
      2. Noworodki urodzone przedwcześnie (wiek ciążowy ≤ 37 tygodni) tak ⁯ nie X
      3. Noworodki (0 - 27 dni) tak ⁯ nie X
      4. Niemowlęta i małe dzieci (28 dni – 23 miesiące) tak ⁯ nie X
      5. Dzieci (2 – 11 lat) tak ⁯ nie X
      6. Młodzież (12 – 17 lat) tak ⁯ nie X
    1. Dorośli (18 – 65 lat) tak X nie ⁯
    2. Osoby starsze(> 65 lat) tak ⁯ nie X
  1. Płeć
     1. Kobiety X
     2. Mężczyźni ⁯
  2. Grupy uczestników badania
     1. Zdrowi ochotnicy tak X nie ⁯
     2. Pacjenci tak ⁯ nie X
     3. Grupy specjalne tak X nie ⁯
        1. Kobiety w wieku rozrodczym tak X nie ⁯
        2. Kobiety w wieku rozrodczym stosujące antykoncepcję tak X nie ⁯
        3. Kobiety ciężarne tak ⁯ nie X
        4. Kobiety karmiące piersią tak ⁯ nie X
        5. Stany nagłe tak ⁯ nie X
        6. Osoby niezdolne do samodzielnego wyrażenia zgody tak ⁯ nie X

Jeżeli „tak”, proszę określić: …

- - - 1. Inne: tak ⁯ nie X

Jeżeli „tak”, proszę wskazać jakie: ………………………

- 1. Planowana liczba uczestników

80 kobiet

- 1. Planowane leczenie lub opieka nad pacjentem lub uczestnikiem badania po zakończeniu jego udziału w badaniu. Proszę wskazać, jeżeli odbiega od zwyczajowego leczenia lub opieki (opis słowny):

Nie dotyczy.

1. **OŚRODKI/BADACZE, KTÓRYCH DOTYCZY NINIEJSZY WNIOSEK**

**Główny Badacz** *(imię i nazwisko, stopień/tytuł naukowy, nazwa jednostki organizacyjnej)*

Dr Natalia Drabińska, Instytut Rozrodu Zwierząt i Badań Żywności PAN w Olsztynie

**Członkowie zespołu badawczego** *(imiona i nazwiska, stopnie/tytuły naukowe, specjalizacja, nazwa jednostki organizacyjnej* )

# dr hab.n.med. Jerzy Romaszko, prof. UWM, Katedra Medycyny Rodzinnej i Chorób Zakaźnych

1. **PODPIS WNIOSKODAWCY** (niniejszym potwierdzam, że zawarte we wniosku dane są zgodne z prawdą) ………………………………………………………………………..

Wniosek do Komisji Bioetycznej

- 1. Data: …
  2. Podpis: …
  3. Imię i nazwisko (drukowanymi literami): ……..
