# Supplementary material for: The effect of isocaloric, energy-restrictive, KETOgenic diet on metabolism, inflammation, nutrition deficiencies and oxidative stress in women with overweight and obesity (KETO-MINOX): Study protocol
Source: PLoS One. 2023 May 8;18(5):e0285283. doi: 10.1371/journal.pone.0285283 (PMC10166534; doi:10.1371/journal.pone.0285283)
Supplement: S2 File — (DOC) [file pone.0285283.s003.doc]

**Application to the Bioethics Committee**

**at the Faculty of Medicine of Collegium Medicum**

**University of Warmia and Mazury in Olsztyn**

**Entitled: Evaluation of the impact of an isocaloric, reducing ketogenic diet on metabolism, inflammation, selected nutritional parameters and oxidative stress in women with overweight and obesity**

**APPLICATION TO THE BIOETHICAL COMMISSION FOR AN OPINION ABOUT**

- clinical trial ⁯

- medical experiment ⁯

- theraphy experiment ⁯

- research experiment ⁯

- scientific research X

1. **APLLICANT**

Institute of Animal Reproduction and Food Research of the Polish Academy of Sciences in Olsztyn, Department of Food Chemistry and Biodynamics, ul. Tuwima 10, 10-748 Olsztyn

University of Warmia and Mazury in Olsztyn, Faculty of Medicine, Collegium Medicum, Department of Family Medicine and Infectious Diseases, ul. Warszawska 30, 10-082 Olsztyn

**Princlipal Investigator**

Name: Natalia Drabińska

Academic degree/title, specialization: Doctor. Leading Field: Agricultural Sciences/Food and Nutrition Technology, Additional Field and Discipline: Medical and Health Sciences/Health Sciences

Phone: +48 733 814 404

E-mail: n.drabinska@pan.olsztyn.pl

**B. CURRENT STATE OF KNOWLEDGE (with references attached)**

Obesity is considered one of the biggest problems of the 21st century. It is defined as an abnormal or excessive accumulation of body fat that increases health risk. The type of obesity is determined on the basis of the body mass index (BMI), calculated as body weight (kg) divided by the square of height (m2), where a BMI of 25 kg/m2 and more is classified as overweight. BMI over 30 kg/m2 is classified as obesity, which can be divided into I (BMI: 30.0 - 34.9 kg/m2), II (BMI: 35.0 - 39.9 kg/m2) and III (BMI: 35.0 - 39.9 kg/m2) : over 40.0 kg/m2) obesity class (Arrone 2002). According to the World Health Organization (WHO), almost 2 billion adults in the world were overweight in 2016, and about 650 million people meet the above-mentioned criterion of obesity. In Europe, it is estimated that up to 50% of the population may have increased body weight (WHO 2016). According to The Global Burden of Disease Study published in 2010, deaths caused by the consequences of obesity are more common than those caused by malnutrition and starvation (Lozano et al., 2012).

The prevalence of obesity and overweight results from the popularity of the so-called Western diet, characterized by excessive consumption of highly processed foods. A caloric surplus diet combined with low physical activity leads to energy imbalance and fat deposition. Importantly, the body protects the body from hunger more effectively than from gaining weight, so it is easier to gain extra pounds than to lose them (Williams et al., 2015). The development of obesity in adulthood may be influenced by environmental, genetic and epigenetic factors already in the prenatal period (Lin et al., 2017). In addition, physio-genetic factors associated with the development of obesity affect caloric intake, thermogenesis, lipid utilization and nutrient turnover (Lin et al., 2017). The endocrine system regulates the feeling of satiety and appetite through adipokines secreted by adipose tissue (Ouchi et al., 2011). Hypertrophy of adipose tissue modifies its secretory status, leading to the development of low-grade inflammation.

Being overweight is not just an aesthetic problem. Increased body weight increases the risk of type 2 diabetes, cardiovascular disease, including hypertension, and certain types of cancer (Williams et al., 2015). The occurrence of obesity-related diseases is closely related to the distribution of adipose tissue, and in particular to the visceral location. The accumulation of visceral fat affects the concentration of adipokines, and consequently contributes to the development of chronic inflammation and metabolic disorders (Graßmann et al., 2017). Adipokines secreted by visceral fat with a role in inflammation, metabolism, or the development of cardiovascular disease include tumor necrosis factor alpha (TNF-a), interleukin-6 (IL-6), IL-beta, adiponectin, leptin, resistin, and serum amyloid A -3 (SAA3), alpha-1 acid glycoprotein, pentraxin-3, IL-1 receptor antagonist, macrophage migration inhibitory factor, plasminogen activator inhibitor-1 (PAI-1), visfatin, vascular endothelial growth factor (VEGF) and many others (Fasshauer and Blüher, 2015; Su and Peng, 2020). Adipokines also induce the production of reactive oxygen species (ROS), generating oxidative stress (Ox) and irregular production of other adipokines (Marseglia et al., 2014). Several mechanisms are involved in the generation of Ox in obesity, and Ox and pro-inflammatory processes are closely related. When activated, many immune cells produce ROS and promote inflammation (Marseglia et al., 2014). Excessive accumulation of fat in obese patients leads to a pathological increase in the level of free fatty acids in the serum, disturbs glucose metabolism, promotes the accumulation of energy substrates in the liver, muscles and adipose tissue (fat and glucose), and promotes the increase in the level of mitochondria and peroxisome oxidation. As a consequence, it leads to the synthesis of free radicals, Ox, mitochondrial DNA damage and finally to lipotoxicity, including various adverse effects of fatty acids on cellular structures (Goossens, 2008). Cell damage leads to high production of cytokines such as TNF-α, which generates further ROS in tissues and increases the rate of lipid peroxidation.

Obesity-induced or co-morbidities, such as metabolic syndrome, respiratory problems and cognitive impairment, have an adverse effect on overall and premature mortality (González-Munies et al., 2017; Jauch-Chara and Oltmanns, 2014; Franks et al. 2010).

Calorie restriction is the most common way to lose weight. Overall, there is no single best nutritional strategy when it comes to sticking to a diet and maintaining energy restrictions (Anton et al., 2017; Johnston et al., 2014). The key issue is not only what type of diet to choose to effectively lose excess weight, but also to avoid the "yo-yo" effect, which will continue after the reduction period. Finally, the diet is supposed to be safe, easy to follow and not causing negative effects. Negative energy balance is critical to diet success (Abete et al., 2010). Many nutritional clinical trials have been conducted on the physiological effects of obesity treatment focused on evaluating different types of diet, including low-calorie (Luglio et al., 2017), very low-calorie (Umphonsathien et al., 2019), high-protein (Benedí et al., 2017) , low-fat diet (Biolato et al., 2019) and intermittent fasting (Liu et al., 2019). Another diet, so far successfully used as an adjuvant therapy in the treatment of epilepsy (Ułamek-Kozioł et al., 2019), is the ketogenic diet (KD). This diet is currently enjoying growing interest as a method of weight reduction. According to a study conducted by the International Food Information Council (IFIC) Foundation with over 1,000 Americans, KD is the only diet with increasing interest (2018-2019) in people motivated by weight loss (https://foodinsight.org). Although the popularity of this diet is growing in the society, knowledge about the safety and effectiveness of KD in weight loss is limited.

The term KD characterizes a high-fat, low-carbohydrate diet that results in a state of ketosis in the body. The idea behind KD was to imitate the physiological state of starvation without fasting. It is worth noting that not all low-carb diets are ketogenic. Ketosis is not observed in a low carbohydrate diet with a high protein intake. This is because many of the amino acids are converted to glucose, which prevents ketosis from occurring (VanItallie & Nufert, 2003).

Previous studies using KD in obesity showed significant reductions in body weight, BMI and body fat after using KD. However, it has not been confirmed that this is due to the state of ketosis, but to a very limited caloric intake. Most of these studies on the use of KD in the treatment of obesity used very low-calorie diets, limited to only 500-800 kcal/day. It can be suspected that the observed changes were due to significantly reduced intake rather than the nutritional composition of the diet, especially since most studies did not include control groups to compare the effects of different dietary compositions (Castro et al., 2018; Hall et al., 2016; Schiavo et al., 2018).

Regarding metabolic effects, very low calorie KD resulted in marked changes in the levels of circulating myokines, including IL-6, IL-8 and metalloproteinase 2, suggesting beneficial health aspects (Sajoux et al., 2019). In addition, low-calorie KD supplemented with DHA reduced the concentration of insulin, triglycerides, total cholesterol, LDL cholesterol, C-reactive protein, TNF-α and resistin (de Luis et al., 2016). The positive effect of very low calorie KD on lipid metabolism and diabetes-related parameters has also been confirmed by other authors (Castaldo et al., 2016; Goday et al., 2016; Ministrini et al., 2019; Pilone et al., 2018). In addition, very low-calorie KD resulted in a reduction in blood pressure (Castaldo et al., 2016; Cicero et al., 2015). However, all these effects were obtained in studies with a very low-calorie KD, dedicated to people qualified for bariatric surgery and observed under clinical supervision. The results of these studies cannot be compared to the effect of KD with an adequate supply of calories or only its slight reduction, which is used by people motivated to lose weight and inspired by social media and influencers to use KD.

Few studies have been reported on KD with optimal caloric intake (Hall et al., 2016; Kenig et al., 2019; Mohorko et al., 2019a; Schiavo et al., 2018). Most of these studies did not include a control group, so the results are difficult to interpret. Considering the studies on KD that included a control group, the positive effect of KD on the treatment of obesity was confirmed in a study of 45 obese women with ovarian or endometrial cancer (Cohen et al., 2018). After twelve weeks, the authors observed a significant loss of visceral, android and total fat mass in the subjects with KD compared to the control group. KD was also associated with lower fasting serum insulin levels in obese women with ovarian or endometrial cancer, the authors suggest by increasing insulin sensitivity (Cohen et al., 2018). Most studies using KD with optimal caloric intake focused solely on changes in body weight and composition and the impact on cardiac parameters (Drabińska et al., 2021). Considering the above, there is a need for comprehensive studies on the physiological effects of KD in overweight people, especially in terms of the presence of inflammation, nutrition and metabolic changes.

On the other hand, it has been suggested that long-term use of KD is associated with the risk of kidney stones, increased blood uric acid levels and osteoporosis (low calcium intake). However, it should be emphasized that long-term AC is mainly used by patients with drug-resistant epilepsy to reduce the frequency of seizures (Ułamek-Kozioł et al., 2019). Diet in people with obesity and overweight is used to reduce weight, hence short dietary interventions are used, usually not lasting longer than a few weeks. The side effects of such interventions are relatively mild and include hunger, fatigue, depressed mood, irritability, constipation, and headaches.

It is also interesting to see if the nutritional composition of the diet affects body weight after the follow-up period, even without close control of the diet in the meantime.

In summary, the available scientific literature indicates that the use of CO may be beneficial in the treatment of obesity, but there is still a lack of comprehensive studies that confirm the effectiveness and safety of the use of CO for weight loss. To date, most studies have had small sample sizes, no control groups, and short-term intervention. Moreover, most of the KD studies were conducted with a very low-calorie KD, and the beneficial effects on body weight could be attributed to the caloric deficit, and not to the nutrient composition of the diet used. The presence of only a few studies conducted on nutritional deficiencies (Kenig et al., 2019) suggests the need to verify data on the intake of minerals, vitamins and nutrients and their concentration in the body.

Taking into account all the information presented above, an intensive and comprehensive analysis of the physiological effects of KD in a randomized and controlled manner seems necessary and justified. The project aims to better understand the metabolic effects of KD in overweight people in a controlled manner, typical of nutritional research - food catering balanced by an experienced dietician, delivered daily to each participant. To the authors' knowledge, there are no completed or ongoing studies focused on the effectiveness of KD in a randomized design, with a properly balanced control group, with a uniform diet administered to all participants throughout the intervention and analyzing multiple parameters simultaneously in one study. The Project will use innovative and efficient techniques and tests to measure the maximum number of parameters from the smallest amount of biological material and in the shortest possible time. The results of the project will directly answer the questions asked by many obese and overweight people who wonder if KD is a safe and effective way to lose weight. The project will make important contributions to food science, dietetics, human nutrition and metabolomics.

**Referenes:**

1. Abete, I., Astrup, A., Martínez, J. A., Thorsdottir, I., & Zulet, M. A. (2010). Obesity and the metabolic syndrome: Role of different dietary macronutrient distribution patterns and specific nutritional components on weight loss and maintenance. Nutrition Reviews, 68(4), 214–231. https://doi.org/10.1111/j.1753-4887.2010.00280.x
2. Anton, S. D., Hida, A., Heekin, K., Sowalsky, K., Karabetian, C., Mutchie, H., Leeuwenburgh, C., Manini, T. M., & Barnett, T. E. (2017). Effects of Popular Diets without Specific Calorie Targets on Weight Loss Outcomes: Systematic Review of Findings from Clinical Trials. Nutrients, 9(8), 822. https://doi.org/10.3390/nu9080822
3. Aronne, L. J. (2002). Classification of Obesity and Assessment of Obesity-Related Health Risks. Obesity Research, 10(S12), 105S-115S. https://doi.org/10.1038/oby.2002.203
4. Benedí, M. V. M., Calahorra, S. P., Sanz, A. M. B., Baila Rueda, L., Lamiquiz Moneo, I., Cenarro, A., Civeira, F., & Gallego, R. M. (2017). A randomized, open-label study to investigate the effect of a high protein diet compared to a normoprotein diet on hydrocarbon metabolism in patients with diabetes or prediabetes and obesity. Atherosclerosis, 263, e263. https://doi.org/10.1016/j.atherosclerosis.2017.06.851
5. Biolato, M., Manca, F., Marrone, G., Cefalo, C., Racco, S., Miggiano, G. A., Valenza, V., Gasbarrini, A., Miele, L., & Grieco, A. (2019). Intestinal permeability after Mediterranean diet and low-fat diet in non-alcoholic fatty liver disease. World Journal of Gastroenterology, 25(4), 509–520. https://doi.org/10.3748/wjg.v25.i4.509
6. Castaldo, G., Monaco, L., Castaldo, L., Galdo, G., & Cereda, E. (2016). An observational study of sequential protein-sparing, very low-calorie ketogenic diet (Oloproteic diet) and hypocaloric Mediterranean-like diet for the treatment of obesity. International Journal of Food Sciences and Nutrition, 67(6), 696–706. https://doi.org/10.1080/09637486.2016.1186157
7. Castro, A. I., Gomez-Arbelaez, D., Crujeiras, A. B., Granero, R., Aguera, Z., Jimenez-Murcia, S., Sajoux, I., Lopez-Jaramillo, P., Fernandez-Aranda, F., & Casanueva, F. F. (2018). Effect of a very low-calorie ketogenic diet on food and alcohol cravings, physical and sexual activity, sleep disturbances, and quality of life in obese patients. Nutrients, 10(10). https://doi.org/10.3390/nu10101348
8. Cicero, A. F. G., Benelli, M., Brancaleoni, M., Dainelli, G., Merlini, D., & Negri, R. (2015). Middle and Long-Term Impact of a Very Low-Carbohydrate Ketogenic Diet on Cardiometabolic Factors: A Multi-Center, Cross-Sectional, Clinical Study. High Blood Pressure and Cardiovascular Prevention, 22(4), 389–394. https://doi.org/10.1007/s40292-015-0096-1
9. Cohen, C. W., Fontaine, K. R., Arend, R. C., Alvarez, R. D., Leath, C. A., Huh, W. K., Bevis, K. S., Kim, K. H., Straughn, J. M., & Gower, B. A. (2018). A ketogenic diet reduces central obesity and serum insulin in women with ovarian or endometrial cancer. Journal of Nutrition, 148(8), 1253–1260. https://doi.org/10.1093/jn/nxy119
10. de Luis, D., Domingo, J. C., Izaola, O., Casanueva, F. F., Bellido, D., & Sajoux, I. (2016). Effect of DHA supplementation in a very low-calorie ketogenic diet in the treatment of obesity: a randomized clinical trial. Endocrine, 54, 111–122. https://doi.org/10.1007/s12020-016-0964-z
11. Drabińska, N., Wiczkowski, W., & Piskuła, M. K. (2021). Recent advances in the application of a ketogenic diet for obesity management. Trends in Food Science and Technology, 110. https://doi.org/10.1016/j.tifs.2021.01.080
12. Fasshauer, M., & Blüher, M. (2015). Adipokines in health and disease. Trends in Pharmacological Sciences, 36(7), 461–470. https://doi.org/10.1016/j.tips.2015.04.014
13. Franks, P. W., Hanson, R. L., Knowler, W. C., Sievers, M. L., Bennett, P. H., & Looker, H. C. (2010). Childhood obesity, other cardiovascular risk factors, and premature death. New England Journal of Medicine, 362(6), 485-493.
14. Goday, A., Bellido, D., Sajoux, I., Crujeiras, A. B., Burguera, B., García-Luna, P. P., Oleaga, A., Moreno, B., & Casanueva, F. F. (2016). Short-Term safety, tolerability and efficacy of a very low-calorie-ketogenic diet interventional weight loss program versus hypocaloric diet in patients with type 2 diabetes mellitus. Nutrition and Diabetes, 6(9), e230. https://doi.org/10.1038/nutd.2016.36
15. González-Muniesa, P., Mártinez-González, M.-A., Hu, F. B., Després, J.-P., Matsuzawa, Y., F Loos, R. J., Moreno, L. A., Bray, G. A., & Alfredo Martinez, J. (2017). Obesity. Nature Reviews, 3, 17034. https://doi.org/10.1038/nrdp.2017.34
16. Goossens, G. H. (2008). The role of adipose tissue dysfunction in the pathogenesis of obesity-related insulin resistance. Physiology & Behavior, 94(2), 206–218. https://doi.org/https://doi.org/10.1016/j.physbeh.2007.10.010
17. Graßmann, S., Wirsching, J., Eichelmann, F., & Aleksandrova, K. (2017). Association Between Peripheral Adipokines and Inflammation Markers: A Systematic Review and Meta-Analysis. Obesity, 25(10), 1776–1785. https://doi.org/10.1002/oby.21945
18. Hall, K. D., Chen, K. Y., Guo, J., Lam, Y., Liebel, R. L., Mayer, L., Reitman, M. L., Rosenbaum, M., Smith, S. R., Walsh, B. T., & Ravussin, E. (2016). Energy expenditure and body composition changes after an isocaloric ketogenic diet in overweight and obese men. American Journal of Clinical Nutrition, 104(2), 324–333. https://doi.org/10.1111/obr.12399
19. Jauch-Chara, K., & Oltmanns, K. M. (2014). Obesity - A neuropsychological disease? Systematic review and neuropsychological model. Progress in Neurobiology, 114, 84–101. https://doi.org/10.1016/j.pneurobio.2013.12.001
20. Johnston, B. C., Kanters, S., Bandayrel, K., Wu, P., Naji, F., Siemieniuk, R. A., Ball, G. D. C., Busse, J. W., Thorlund, K., Guyatt, G., Jansen, J. P., & Mills, E. J. (2014). Comparison of Weight Loss Among Named Diet Programs in Overweight and Obese Adults: A Meta-analysis. JAMA, 312(9), 923–933. https://doi.org/10.1001/jama.2014.10397
21. Kenig, S., Petelin, A., Poklar Vatovec, T., Mohorko, N., & Jenko-Pražnikar, Z. (2019). Assessment of micronutrients in a 12-wk ketogenic diet in obese adults. Nutrition, 67–68, 2–8. https://doi.org/10.1016/j.nut.2019.06.003
22. Lin, X., Lim, I. Y., Wu, Y., Teh, A. L., Chen, L., Aris, I. M., Soh, S. E., Tint, M. T., MacIsaac, J. L., Morin, A. M., Yap, F., Tan, K. H., Saw, S. M., Kobor, M. S., Meaney, M. J., Godfrey, K. M., Chong, Y. S., Holbrook, J. D., Lee, Y. S., … group, G. study. (2017). Developmental pathways to adiposity begin before birth and are influenced by genotype, prenatal environment and epigenome. BMC Medicine, 15(1), 50. https://doi.org/10.1186/s12916-017-0800-1
23. Liu, B., Hutchison, A. T., Thompson, C. H., Lange, K., & Heilbronn, L. K. (2019). Markers of adipose tissue inflammation are transiently elevated during intermittent fasting in women who are overweight or obese. Obesity Research and Clinical Practice, 13(4), 408–415. https://doi.org/10.1016/j.orcp.2019.07.001
24. Lozano, R., Naghavi, M., Foreman, K., Lim, S., Shibuya, K., Aboyans, V., Abraham, J., Adair, T., Aggarwal, R., Ahn, S. Y., AlMazroa, M. A., Alvarado, M., Anderson, H. R., Anderson, L. M., Andrews, K. G., Atkinson, C., Baddour, L. M., Barker-Collo, S., Bartels, D. H., … Murray, C. J. L. (2012). Global and regional mortality from 235 causes of death for 20 age groups in 1990 and 2010: A systematic analysis for the Global Burden of Disease Study 2010. The Lancet, 380, 2095–2128. https://doi.org/10.1016/S0140-6736(12)61728-0
25. Luglio, H. F., Sulistyoningrum, D. C., Muharomin, I. R., & Huriyati, E. (2017). Leptin, appetite and weight rebound in overweight/obesity individuals undertook weight loss program using a low calorie diet with or without exercise. Mediterranean Journal of Nutrition and Metabolism, 10(3), 223–233. https://doi.org/10.3233/mnm-17162
26. Marseglia, L., Manti, S., D’Angelo, G., Nicotera, A., Parisi, E., Di Rosa, G., Gitto, E., & Arrigo, T. (2014). Oxidative stress in obesity: a critical component in human diseases. International Journal of Molecular Sciences, 16(1), 378–400. https://doi.org/10.3390/ijms16010378
27. Ministrini, S., Calzini, L., Nulli Migliola, E., Ricci, M. A., Roscini, A. R., Siepi, D., Tozzi, G., Daviddi, G., Martorelli, E.-E., Paganelli, M. T., & Lupattelli, G. (2019). Lysosomal Acid Lipase as a Molecular Target of the Very Low Carbohydrate Ketogenic Diet in Morbidly Obese Patients: The Potential Effects on Liver Steatosis and Cardiovascular Risk Factors. Journal of Clinical Medicine, 8, 621. https://doi.org/10.3390/jcm8050621
28. Mohorko, N., Černelič-Bizjak, M., Poklar-Vatovec, T., Grom, G., Kenig, S., Petelin, A., & Jenko-Pražnikar, Z. (2019a). Weight loss, improved physical performance, cognitive function, eating behavior, and metabolic profile in a 12-week ketogenic diet in obese adults. Nutrition Research, 62, 64–77. https://doi.org/10.1016/j.nutres.2018.11.007
29. Mohorko, N., Černelič-Bizjak, M., Poklar-Vatovec, T., Grom, G., Kenig, S., Petelin, A., & Jenko-Pražnikar, Z. (2019b). Weight loss, improved physical performance, cognitive function, eating behavior, and metabolic profile in a 12-week ketogenic diet in obese adults. Nutrition Research, 62, 64–77. https://doi.org/10.1016/j.nutres.2018.11.007
30. Ouchi, N., Parker, J. L., Lugus, J. J., & Walsh, K. (2011). Adipokines in inflammation and metabolic disease. Nature Reviews Immunology, 11(2), 85–97. https://doi.org/10.1038/nri2921
31. Pilone, V., Tramontano, S., Renzulli, M., Romano, M., Cobellis, L., Berselli, T., & Schiavo, L. (2018). Metabolic effects, safety, and acceptability of very low-calorie ketogenic dietetic scheme on candidates for bariatric surgery. Surgery for Obesity and Related Diseases, 14(7), 1013–1019. https://doi.org/10.1016/j.soard.2018.03.018
32. Sajoux, I., Lorenzo, P. M., Gomez-Arbelaez, D., Zulet, M. A., Abete, I., Castro, A. I., Baltar, J., Portillo, M. P., Tinahones, F. J., Martinez, J. A., Crujeiras, A. B., & Casanueva, F. F. (2019). Effect of a Very-Low-Calorie Ketogenic Diet on Circulating Myokine Levels Compared with the Effect of Bariatric Surgery or a Low-Calorie Diet in Patients with Obesity. Nutrients, 11(10). https://doi.org/10.3390/nu11102368
33. Schiavo, L., Pilone, V., Rossetti, G., Barbarisi, A., Cesaretti, M., & Iannelli, A. (2018). A 4-Week Preoperative Ketogenic Micronutrient-Enriched Diet Is Effective in Reducing Body Weight, Left Hepatic Lobe Volume, and Micronutrient Deficiencies in Patients Undergoing Bariatric Surgery: a Prospective Pilot Study. Obesity Surgery, 28(8), 2215–2224. https://doi.org/10.1007/s11695-018-3145-8
34. Su, X., & Peng, D. (2020). Adipokines as novel biomarkers of cardio-metabolic disorders. Clinica Chimica Acta, 507, 31–38. https://doi.org/https://doi.org/10.1016/j.cca.2020.04.009
35. Ułamek-Kozioł, M., Czuczwar, S. J., Januszewski, S., & Pluta, R. (2019). Ketogenic Diet and Epilepsy. Nutrients, 11(10), 2510. https://doi.org/10.3390/nu11102510
36. Umphonsathien, M., Prutanopajai, P., Aiam-O-Ran, J., Thararoop, T., Karin, A., Kanjanapha, C., Jiamjarasrangsi, W., & Khovidhunkit, W. (2019). Immediate and long-term effects of a very-low-calorie diet on diabetes remission and glycemic control in obese Thai patients with type 2 diabetes mellitus. Food Science and Nutrition, 7(3), 1113–1122. https://doi.org/10.1002/fsn3.956
37. VanItallie, T. B., & Nufert, T. H. (2003). Ketones : Metabolism ’ s Ugly Duckling. Nutrition Reviews, 61(10), 327–341. https://doi.org/10.131/nr.2003.oct.327
38. WHO. (2016). Obesity and overweight: Fact sheet. In WHO Media Centre.
39. Williams, E. P., Mesidor, M., Winters, K., Dubbert, P. M., & Wyatt, S. B. (2015). Overweight and Obesity: Prevalence, Consequences, and Causes of a Growing Public Health Problem. Current Obesity Reports, 4(3), 363–370. https://doi.org/10.1007/s13679-015-0169-4
    1. PLEASE INDICATE THE CONDITION(S) OR DISEASE(S) THAT ARE THE SUBJECT OF THE EXAMINATION (WORDS)

# Obesity and overweight

- 1. Is it a rare disease? yes ⁯ no X
  2. Area of research: (mar kall suitable)
     1. Diagnostic ⁯
     2. Prophylaxis X
     3. Therapy ⁯
     4. Safety X
     5. Effectiveness X
     6. Pharmacokinetics ⁯
     7. Pharmacodynamics ⁯
     8. Bioequivalence ⁯
     9. Dose-response relationship ⁯
     10. Pharmacogenetics ⁯
     11. Pharmacogenomics ⁯
     12. Pharmacoeconomics ⁯
     13. Other ⁯

If „other”, please name it:…………………………………

1. **AIMS OF STUDY**
   1. Main aim: …

The primary goal is to evaluate the impact of using a ketogenic diet as a weight loss diet on inflammation and oxidative stress in overweight and obese women

- 1. Secondary aims:

# Secondary aims:

1) assessment of the impact of the ketogenic diet on body mass and composition

2) assessment of the impact of weight loss associated with the ketogenic diet on the nutritional status of overweight and obese women

3) assessment of the impact of weight loss associated with the ketogenic diet on the profile of metabolites in exhaled air,

4) assessment of the impact of weight loss associated with the ketogenic diet on parameters related to obesity and diabetes, including lipid profile, adipokine status

5) assessment of the long-term effects and effectiveness of the ketogenic diet

6) assessment of the impact of the ketogenic diet on the state of the intestinal microbiota

- 1. Is there substudies? yes ⁯ no X

If yes, please provide the full title, date and version of each sub-study and their association with the main study: ………………………………………………………………………..

**C. MAIN INCLUSION CRITERIA (please list the most important):**

Women aged 18-45 (in childbearing years), using hormonal or barrier contraception during the study, BMI 25.5 - 35, motivation to reduce weight and participate in the study

**MAIN EXCLUSION CRITERIA (please list the main ones):**

Overweight/obesity resulting from genetic diseases, endocrine, autoimmune, psychiatric diseases, pregnancy, breastfeeding, diabetes, other chronic diseases requiring pharmacotherapy, participation in other clinical trials, type II and III obesity (BMI > 35), weight loss > 3 kg in the 12 weeks before the start of the study, large changes in exercise intensity in the 4 weeks before the start of the study

1. **PRIMARY OUTCOMES:**

The main endpoint of the study here is the change in the body weight of the volunteer on the day of the end of the diet. In addition, the following will be assessed: the levels of lipid parameters and blood count, the level of cytokines and parameters related to the development of obesity and diabetes, oxidative stress parameters, the concentration of fat-soluble vitamins and amino acids, breath metabolites and intestinal microbiota after the end of the diet and 12 months after the end of the diet.

1. **STUDY DESIGN**
   1. Controlled yes X no ⁯

If yes, please mark: …

- - 1. Randomized yes X no ⁯
    2. Open yes X no ⁯
    3. Single blind yes ⁯ no X
    4. Double blind yes ⁯ no X
    5. Parallel groups yes X no ⁯
    6. Cross-sectional yes ⁯ no X
    7. Prospective yes X no ⁯
    8. Retrospective yes ⁯ no X
    9. Other yes ⁯ no X

If other, please indicate: …

- 1. Side effects (załącznik nr 3)
     1. Methods of recording side effects

Side effects, if any, will be recorded by participants in the study diary.

- - 1. Procedures in the event of complications.

Participants in the study who are suspected of having an adverse effect of the diet on their health will undergo a medical assessment (Jerzy Romaszko, Sebastian Borowicz-Skoneczny), and if confirmed, will be excluded from the study. Mild gastric complaints resulting from a change in diet are not an indication for exclusion from the study. Illnesses resulting from other causes will be treated within the health care system. Depending on their nature, they may or may not be grounds for exclusion from the study. Each such case will be assessed individually.

- 1. Definition of the end of study:

The study will be completed 12 months after the end of the diet. Each participant may discontinue participation in the study at any time.

- 1. Initial estimated study plan:

Expected start date of the study (day, month, year) April 1, 2023

Expected date of completion of the study (day, month, year) June 1, 2024

- 1. ACCORDING TO THE RESOLUTION, THE RESEARCHER SUBMITS A REPORT ON THE EXAMINATION WITHIN THE DATE SPECIFIED IN THE RESOLUTION.

1. **Patient groups**
   1. Age groups
      1. Below 18 yes ⁯ no X

If yes:

- - - 1. Intrauterine development yes ⁯ no X
      2. Preterm neonates yes ⁯ no X
      3. Newbornes (0 - 27 days) yes ⁯ no X
      4. Infants and young children (28 days – 23 months) yes ⁯ no X
      5. Children (2 – 11 y) yes ⁯ no X
      6. Adolescents (12 – 17 y) yes ⁯ no X
    1. Adults (18 – 65 y) yes X no ⁯
    2. Elderly (> 65 y) yes ⁯ no X
  1. Gender
     1. Women X
     2. Men ⁯
  2. Participant groups
     1. Heathy individuals yes X no ⁯
     2. Patients yes ⁯ no X
     3. Special groups yes X no ⁯
        1. Women in reproductive age yes X no ⁯
        2. Women in reproductive age which use contraceptives yes X no ⁯
        3. Pregnant women yes ⁯ no X
        4. Breastfeeding women yes ⁯ no X
        5. Emergencies yes ⁯ no X
        6. Persons incapable of giving consent on their own yes ⁯ no X

In yes, specify: …

- - - 1. Other: yes ⁯ no X

If yes, specify: ………………………

- 1. Planned number of participants

80

- 1. PLANNED TREATMENT OR CARE OF THE PATIENT OR STUDY PARTICIPATION AFTER PARTICIPATION IN THE STUDY. PLEASE INDICATE IF IT DIFFERS FROM USUAL TREATMENT OR CARE (WRITTEN DESCRIPTION):

Not applicable

1. **RESEARCHERS INVOLVED IN THE PROJECT**

**Principal investigator** *(name and surname, academic degree/title, name of the organizational unit)*

Dr Natalia Drabińska, Institute of Animal Reproduction and Food Research of the Polish Academy of Sciences in Olsztyn

**Team members** *(name and surname, academic degree/title, name of the organizational unit)*

**dr hab.n.med. Jerzy Romaszko, prof. UWM, Department of Family Medicine and Infectious Diseases**

1. **Signatures** (I hereby confirm that the information provided in the application is true) ………………………………………………………………………..

Application to the Ethical Comeetee

- 1. Date: …
  2. Signature: …
  3. Name: ……..
